# In silico and in vitro investigations reveal pan-PPAR agonist activity and anti-NAFLD efficacy of polydatin by modulating hepatic lipid-energy metabolism

**DOI:** 10.1038/s41598-025-12357-0

**Published:** 2025-07-24

**Authors:** Sumit Kumar Mandal, Mohammed Muzaffar-Ur-Rehman, Sonakshi Puri, Pankaj Kumar Sharma, Sankaranarayanan Murugesan, P. R. Deepa

**Affiliations:** 1 Biochemistry and Enzyme Biotechnology Laboratory, Department of Biological Sciences, Birla Institute of Technology and Science Pilani, Pilani Campus, Pilani, Rajasthan 333 031 India; 2 Medicinal Chemistry Research Laboratory, Department of Pharmacy, Birla Institute of Technology and Science Pilani, Pilani Campus, Pilani, Rajasthan 333 031 India

**Keywords:** Peroxisome proliferator-activated receptors (PPARs), Pan-agonist, Drug repurposing, NAFLD, Resveratrol, Polydatin, Computational biology and bioinformatics, Molecular biology, Drug screening, Target identification

## Abstract

Polydatin (PD), a stilbenoid resveratrol-derivative in *Vitaceae*,* Liliaceae*,* and Leguminosae*, exhibits pharmacological protection in metabolic disorders. This study investigated Polydatin, as a potential pan-PPAR agonist for treating non-alcoholic fatty liver disease (NAFLD). High-throughput-virtual-screening (HTVS) was performed to identify potential pan-PPAR agonists, followed by in vitro testing of Polydatin in HepG2 steatosis model. Effects on lipid metabolism and oxidative stress, PPAR signaling gene expression analysis, and GC-MS profiling were compared with the hepatoprotectant Silymarin. Pan-PPAR targeted HTVS of PhytoHub natural products database, followed by molecular docking/dynamics simulations, revealed lead-candidate, Polydatin, which was tested in steatotic cells for gene and protein deregulations by qRT-PCR and western blot, followed by GC-MS analysis of biochemical metabolites. HTVS revealed 53 potential pan-PPAR agonists. Molecular docking and dynamics simulations suggested that PD, a stable ligand for PPARs (α,β/δ,γ), exhibited strong binding. Polydatin treatment decreased ALT, triglycerides, and oxidative stress, wherein ROS and malondialdehyde levels decreased by 60.94% and 28%, respectively. PD upregulated PPARs, AMPK, GLUT2, and CPT1α, while downregulating lipogenic enzymes (ACC1, FASN, SCD1). GC-MS analysis revealed Polydatin mediated impact on saturated FFAs-palmitic acid, stearic acid, and unsaturated fatty acid product of SCD1, oleic acid. HTVS identified PD as a promising pan-PPAR agonist, which favorably ameliorated changes in lipid, glucose, and overall energy metabolism in steatotic NAFLD, by modulating PPAR(α,β/δ,γ) expressions and associated downstream lipogenic and lipid-utilization mechanisms, supporting anti-steatotic efficacy of Polydatin.

**Supplementary Information:**

The online version contains supplementary material available at 10.1038/s41598-025-12357-0.

## Introduction

The lipid-sensing nuclear receptors, Peroxisome-proliferator-activated receptors (PPARs), regulate glucose and lipid metabolisms, which are crucial for maintaining energy homeostasis via their trans-activation or trans-repression changes^1^. The functional impairment/dysregulation of PPARs causes disease progression and complications in metabolic disorders^2,3^. The PPAR family of receptors has gained prominence in recent years for therapeutic targeting in metabolic syndrome (MetS)^4^. PPARs have three isoforms α, β/δ, and γ^5^. They are crucial for energy metabolism but have different functions: PPAR-γ is primarily involved in energy storage, while PPAR-α is mainly found in the liver and, to a lesser extent, in muscle, heart, and bone. PPAR-δ, on the other hand, is widely distributed throughout the body and plays a role in regulating energy expenditure. Additionally, PPAR-γ is expressed in endothelial cells and vascular smooth muscle cells^6,7^.

Current treatments for metabolic disorders using single or dual PPAR agonists are limited by safety concerns like weight gain, fluid retention, inflammation, and potential tumor development^8^. To address these issues, pan-PPAR agonists, which activate all three PPAR receptors, are being explored. This approach aims to provide broader therapeutic benefits while mitigating the side effects associated with selective agonists^9^. Promisingly, Lanifibranor, targeting liver fibrosis, and Chiglitazar, for type 2 diabetes, have both demonstrated positive results in recent phase 3 clinical trials^10^.

Recent research shows that phytochemical-rich foods may reduce both hyperinsulinemia and insulin resistance^11,12^. A clinical study revealed that consuming flavonoids lowers the risk of non-alcoholic fatty liver disease (NAFLD) and helps normalize its condition by reducing the fatty liver index and levels of serum aspartate aminotransferase and alanine aminotransferase^13^. Some studies suggested that catechin, which is one of the main flavonoids of green tea, improves insulin resistance in patients suffering from NAFLD^14^. Green, black, and dark teas have been associated with improved liver function through the repression of reactive oxygen species, inflammatory cytokines, and modulation of glucose and lipid metabolism^15^. Polyphenols are the main antioxidants found in our diet, and their effects are central to the widely recognized health advantages of fruits and vegetables to various diseases such as neurodegenerative diseases, cardiovascular disease, osteoporosis, cancer, inflammatory disorders, diabetes, and Infectious illnesses^16–18^. Additionally, there is evidence suggesting that these substances may affect the process of de novo lipogenesis^16,19^.

*Silybum marianum*, commonly known as milk thistle, has been recognized since ancient times for its potential benefits in treating liver diseases. The active compound in milk thistle, Silymarin, consists of four isomer flavonolignans. Its positive impact on liver health is believed to come from its anti-inflammatory, antioxidant, and antifibrotic properties^20,21^. Additionally, silymarin may help reduce insulin resistance, and various studies on different liver conditions have demonstrated its efficacy and excellent safety profile^22^.

Resveratrol, a polyphenol found in grapes, red wine, peanuts, and berries, demonstrated potential in reducing liver enzymes (AST and ALT), fasting blood sugar levels, and insulin resistance^23^. There are reports of favorable effects of Polydatin in various diseases such as cancers, cardiovascular diseases, and diabetes^24,25^, hepatic and respiratory disorders^26^, gastrointestinal ailments^27^, infectious diseases^28^, and rheumatoid disorders^29^. Recently, anthocyanins from sweet cherries have also been elucidated for their benefits in decreasing liver fat accumulation through inhibition of lipogenesis and promotion of lipolysis in NAFLD^30^.

In the present study, the outcomes of pan-PPAR targeted HTVS of Phytohub database of natural products has been presented. One of the top-scoring ligands was Polydatin (trans piceid), which was further experimentally validated in vitro for its anti-NAFLD activity and biochemical mechanisms.

## Results

### Molecular docking analysis

A total of 1200 compounds have been docked in the active sites of PPARα, PPARγ, and PPARβ/δ. To begin with, the hits were screened by HTVS, allowed to select top scoring ligands as well as to conduct further screenings in SP mode. Those compounds that did not bind in the SP mode were again discarded, and the rest of the compounds underwent the XP mode docking. These resulted in 53 compounds that showed optimal binding scores with all the three PPAR isoforms (Supplementary Table 1). Further, the results for each of the three isoforms were combined to find the ligands that shows the pan-PPAR agonist docking scores. The list of top 25 pan-PPAR ligands with respect to the binding energies are reported in Table 1.

**Table 1:** Docking scores of the top ligands against the active sites of PPARα, β/δ, γ.

		2ZNN (PPARα)	3GZ9 (PPARβ/δ)	2ATH (PPARγ)
1	Demethyloleuropein	− 12.209	− 13.126	− 14.516
2	Eriodictyol-7-glucuronide	− 13.046	− 12.049	− 12.375
3	5-(3ʹ-hydroxyphenyl)-gamma-valerolactone-4ʹ-*O*-beta-d-glucuronide	− 11.424	− 12.379	− 12.023
4	cis-Resveratrol-3-*O*-glucuronide	− 12.112	− 11.013	− 11.942
5	Protocatechuic acid-3-*O*-glucuronide	− 9.976	− 10.762	− 11.877
6	6-Hydroxyluteolin 7-*O*-rhamnoside	− 12.186	− 12.662	− 11.819
7	(−)-Epicatechin 4ʹ-*O*-glucuronide	− 10.342	− 11.031	− 11.759
8	(−)-Epicatechin-5-*O*-glucuronide	− 11.871	− 12.064	− 11.710
10	Farnesol glucuronide	− 10.555	− 11.585	− 11.604
11	Luteolin 7-*O*-glucoside	− 11.508	− 11.104	− 11.603
12	3-(3?-Hydroxyphenyl)propionic acid-glucuronide	− 10.576	− 10.829	− 11.553
13	Dihydro-resveratrol-3-*O*-glucuronide	− 11.759	− 11.582	− 11.500
14	3-Phenylpropionic acid-4ʹ-*O*-glucuronide	− 8.032	− 10.783	− 11.475
15	Dihydro-piceid	− 11.392	− 11.898	− 11.460
16	cis-Resveratrol-4ʹ-*O-*glucuronide	− 11.034	− 11.849	− 11.444
17	Isourolithin A-3-glucuronide	− 10.556	− 9.287	− 11.428
18	Perillic acid glucuronide	− 8.686	− 10.543	− 11.198
19	Scutellarin	− 12.842	− 8.853	− 11.126
20	3-*O*-Caffeoylquinic acid	− 10.136	− 9.661	− 11.057
21	5-*O*-Caffeoylquinic acid	− 10.136	− 9.661	− 11.057
22	Neochlorogenic acid	− 10.136	− 9.661	− 11.057
23	Resveratrol-3-*O*-glucuronide	− 11.628	− 11.258	− 11.044
24	trans-resveratrol-5-*O*-glucuronide	− 11.628	− 11.258	− 11.044
25	(+)-Catechin 3-*O*-glucose	− 10.582	− 11.940	− 11.015

The above table presents the top 25 molecules with high binding affinity with all three PPAR isoforms. A total list of 53 such compounds were identified, which have been compiled in Supplementary Table 1.

A few factors were considered in order to select one ligand for further experimental validation. Table 1 was compiled based on the ligand affinity that was optimal for all the three isoforms of PPAR, in order to qualify as pan-agonist. From this list of about 53 ligands, we excluded the top-scoring demethyloleuropein, and other glucuronide ligands in the list for the following reasons. The compounds resulting from glucuronidation become highly hydrophilic and cannot pass through the cell membrane via passive diffusion. Instead, these metabolites require active efflux from the cells that are facilitated by a variety of efflux transporters^31^. Therefore, the glucuronides compounds were not selected for further in vitro study here, as the target was nuclear receptor located within the cells. The highest scoring ligand, demethyloleuropein, showed greater variation in its energy scores between the PPAR isoforms. Therefore, the next non-glucuronide ligand molecule, dihydro-piceid was considered. However, Piceid has several chemical analogues. Amongst these, trans-piceid, also known as Polydatin, is emerging as a nutraceutical with beneficial biological properties. Therefore, Polydatin was subjected to docking analysis, which yielded favorable Glide scores as well as a balanced energy profile (Supplementary Fig. 1) across the three PPAR isoforms. Polydatin was then subjected to additional confirmatory in silico simulations and pharmacokinetic analysis, followed by in vitro pharmacological validations.

### In Silico ADMET and drug-likeness analysis

All the ligands showed acceptable scores for polarity, hydrophobicity, and lipophilicity. Drug-likeness values were obtained based on the topological polar surface area (TPSA) and partition coefficient (LogP). Table 2 summarizes the in silico predicted ADMET characteristic results for the top pan-PPAR agonists. ADMET characteristics are interpreted based on specific values, such as high Caco-2 permeability predicted value > 0.90, intestinal absorption less than 30% is considered poorly absorbed, low human VDss if less than 0.71 L/kg and high if more than 2.81 L/kg, BBB permeability logBB > 0.3 considered to cross BBB, and logBB 1 indicating poor distribution. CNS permeability is measured by logPS > 2 for permeation into the CNS, while logPS 3 indicates inability.

**Table 2:** In silico predicted pharmacokinetic (ADMET) analysis of the top-scoring ligands.

Parameters	Absorption						Distribution			
	Water solubility (log mol/L)	Caco2 permeability (log cm/s)	Intestinal absorption (%human)	Skin Permeability (log Kp)	*P*-glycoprotein substrate	*P*-glycoprotein inhibitor	VDss (human) (log L/kg)	Fraction unbound (human) (Fu)	BBB permeability (log BB)	CNS permeability (log PS)
Dihydro-piceid	0.974	− 4.89	0.974	− 2.39	Yes	No	1.5	0.74	− 1.74	No
Cis/ trans Piceid	− 2.90	− 6.56	0.608	− 1.44	Yes	Yes	0.86	0.98	− 3.15	No
Demethyl-oleuropein	0.338	− 6.39	0.338	− 3.35	Yes	No	0.64	0.86	− 3.33	No
Oleuropein	0.532	− 6.38	0.532	− 0.72	Yes	No	0.69	0.78	− 3.56	No

Parameters	Excretion		Toxicity			Lipinski’s rule of five					
	Total Clearance (log ml/min/kg)	Renal OCT2 substrate	AMES toxicity	Hepatotoxicity	Skin sensitization	Molecular weight	LogP	Rotatable bonds	Acceptors	Donors	Surface area
Dihydro-piceid	2.99	No	No	Yes	Yes	230.263	2.5886	3	3	3	99.601
Cis/ trans Piceid	11.66	No	No	No	No	390.388	0.4469	5	8	6	160.705
Demethyl-oleuropein	6.38	No	No	No	Yes	526.491	− 0.7226	9	12	7	210.946
Oleuropein	9.62	No	No	No	No	928.89	− 0.4366	15	21	11	376.339

*Caco2* Cancer coli-2, *BBB* Blood–brain barrier, *VDss* Volume of distribution at steady state, *CNS* central nervous system, *OCT2* Organic Cation Transporter 2, *CYP* Cytochromes P450.

The favourable pharmacokinetic profile of these ligands is yet another point for considering these ligands as potential PPAR pan-agonist drug candidates. Among the five, Polydatin had a better predicted pharmacokinetic profile, which confirmed our selection criteria to carry it forward for further biochemical studies.

### Molecular dynamics analysis

**Fig. 1:**
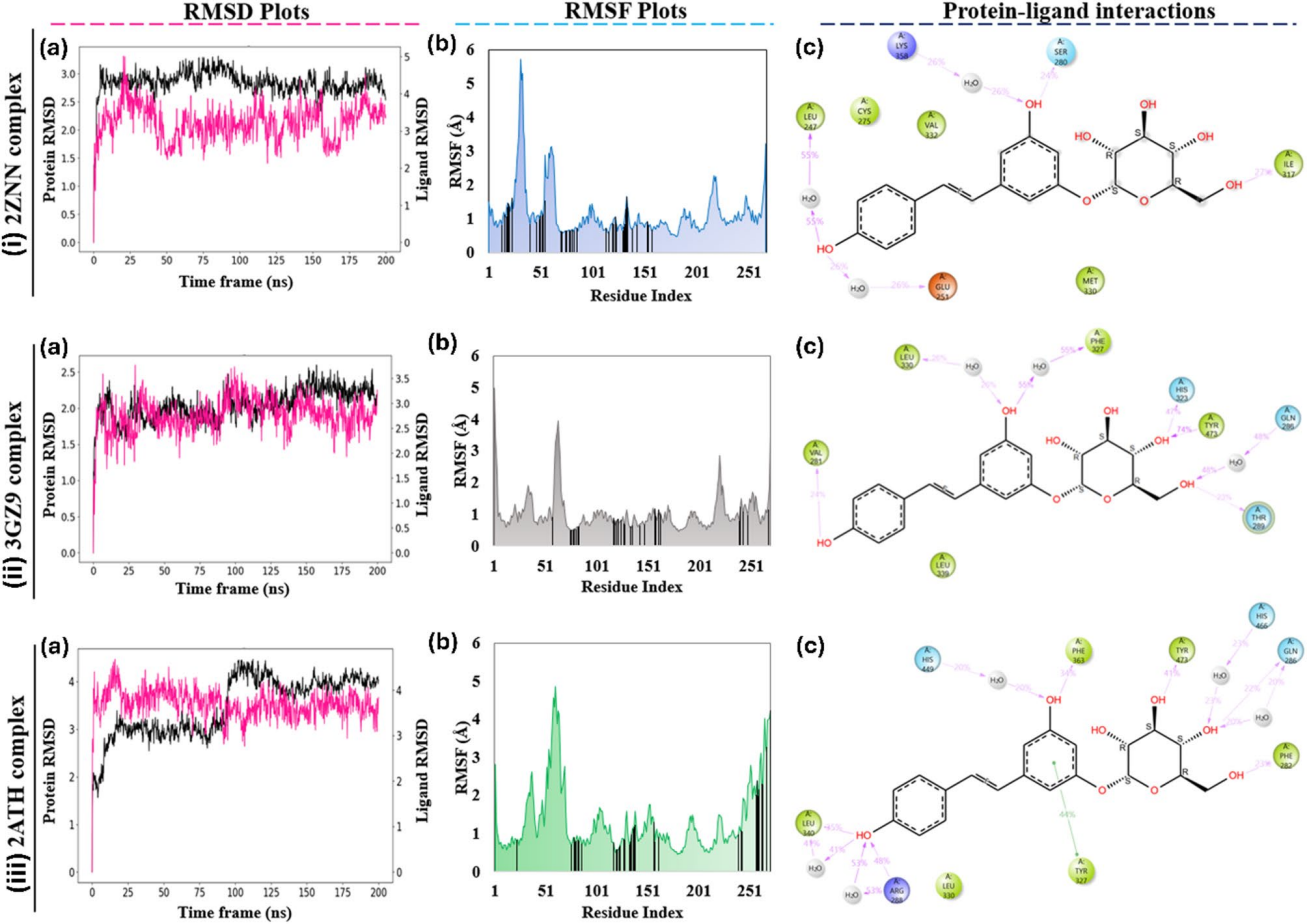
Molecular dynamics simulation analysis of Polydatin binding to pan-PPAR isoforms. Panels (i–iii) represent results for the three PPAR isoforms: PPARα (i, 2ZNN complex), PPARβ/δ (ii, 3GZ9 complex), and PPARγ (iii, 2ATH complex). (**a**) Root Mean Square Deviation (RMSD) plots showing protein (black) and ligand (magenta) stability over a 200 ns simulation timeframe. (**b**) Root Mean Square Fluctuation (RMSF) plots representing the residue-wise atomic fluctuations of the protein backbone. (**c**) 2D interaction diagrams showing key protein–ligand interactions for Polydatin with the respective PPAR isoform, highlighting hydrogen bonds, hydrophobic interactions, and water bridges.

The molecular dynamic simulations for Polydatin across pan-PPAR targets were performed 200 ns using the Desmond module of the Schrödinger software (Schrödinger LLC., NY, v2020). From the RMSD plot of PPARα (2ZNN) complex, it shows stable protein deviations ranging between 2.5 Å and 3.2 Å, while the ligand showed initial adjustment for around 30 ns, and thereafter, showed deviations in the range of 2.4 Å and 4.2 Å. Since both the protein and ligand deviations shows less than 2.0 Å range difference, the complex is said to be stable (Fig. 1ia). Moreover, the RMSF plot of the PPARα shows less fluctuations of the interacting residues (< 1.8 Å), indicating minimal mobility of the binding site residues (Fig. 1ib). The ligand, Polydatin shows moderate water-mediated H-bond interaction (25-55%) with Leu247, Glu251, and Lys358, while Ser280 and Ile317 shows direct H-bond interactions. Several other interactions with < 10% contributions are also seen (Fig. 1ic). For the PPARβ (3GZ9) complex, the protein RMSD is between the range of 1.8 Å to 2.7 Å while its RMSF of the interacting residues were < 1.8 Å indicating limited flexibility. The ligand RMSD is in the range of 2.0 Å to 3.6 Å suggesting that the ligand remained consistently associated with the proteins’ active site residues for the entire simulation (Fig. 1iia,b). Direct H-bond interactions are seen by the residues Tyr473 and His23 with 74% and 47% respectively, while two water-mediated interactions are seen each with Gln286 (48%) and Phe327 (55%) (Fig. 1iic. In the case of PPARγ (2ATH) complex, the protein deviated between 3.0 Å to 4.5 Å, while the ligand adjusted initially for 20 ns, thereafter, remained stable for 100 ns and later increased slightly to 4.2 å and remained stable until the end (Fig. 1iiia). The interacting residues has fluctuations < 1.8 Å except for terminal residues which deviated up to 4.0 Å (Fig. 1iiib). There are residues that contributed moderately in the interaction during the simulation which include Phe282, Arg288, Phe363, Leu340 and Tyr473 (23–48%) via H-bond and Tyr327 (44%) via π–π stacking interaction. A few water-mediated interactions are also observed with Gln286, Arg88, Leu340, His466 and His449 (20-53%) (Fig. 1iiic).

To observe the behaviour of the proteins in the unbound state (apo-forms), we also carried out molecular dynamics simulations for 200 ns on the apo-forms of the PPARα, β, and γ protein. Analysis of the RMSD plot revealed that the α-form shows deviations ranging from 2.0 Å to 4.0 Å, while the β-form ranges between 1.4 Å to 3.0 Å and the γ-form between 1.5 Å and 3.2 Å. These deviations are relatively higher compared to their corresponding ligand-bound complexes. Furthermore, the RMSF plot of the apo forms also shows slightly greater fluctuations in the active site residues suggesting increased flexibility in the absence of the Polydatin. The observations indicate that the apo forms of the proteins have higher variability in their RMSD and RMSF profiles, while corresponding complexes shows stabilizing effect of the protein when bound with the Polydatin (Supplementary Fig. 2a–f). Moreover, the radius of gyration (rGyr) determines how compact the complex structure is during the simulation, and for all the three complexes, the rGyr was between 4.5 Å and 5.4 Å (Supplementary Fig. 3). Therefore, the RMSD plot with lower deviation (< 2.0 Å), the RMSF plot showing lower residual fluctuations (< 1.8 å), and the radius of gyration (< 1.0 Å) indicate that all three complexes are stable during the complete simulation.

### Biochemical analysis of top ligand, polydatin

#### Evaluation of the toxicity of polydatin in HepG2 cells

**Fig. 2:**
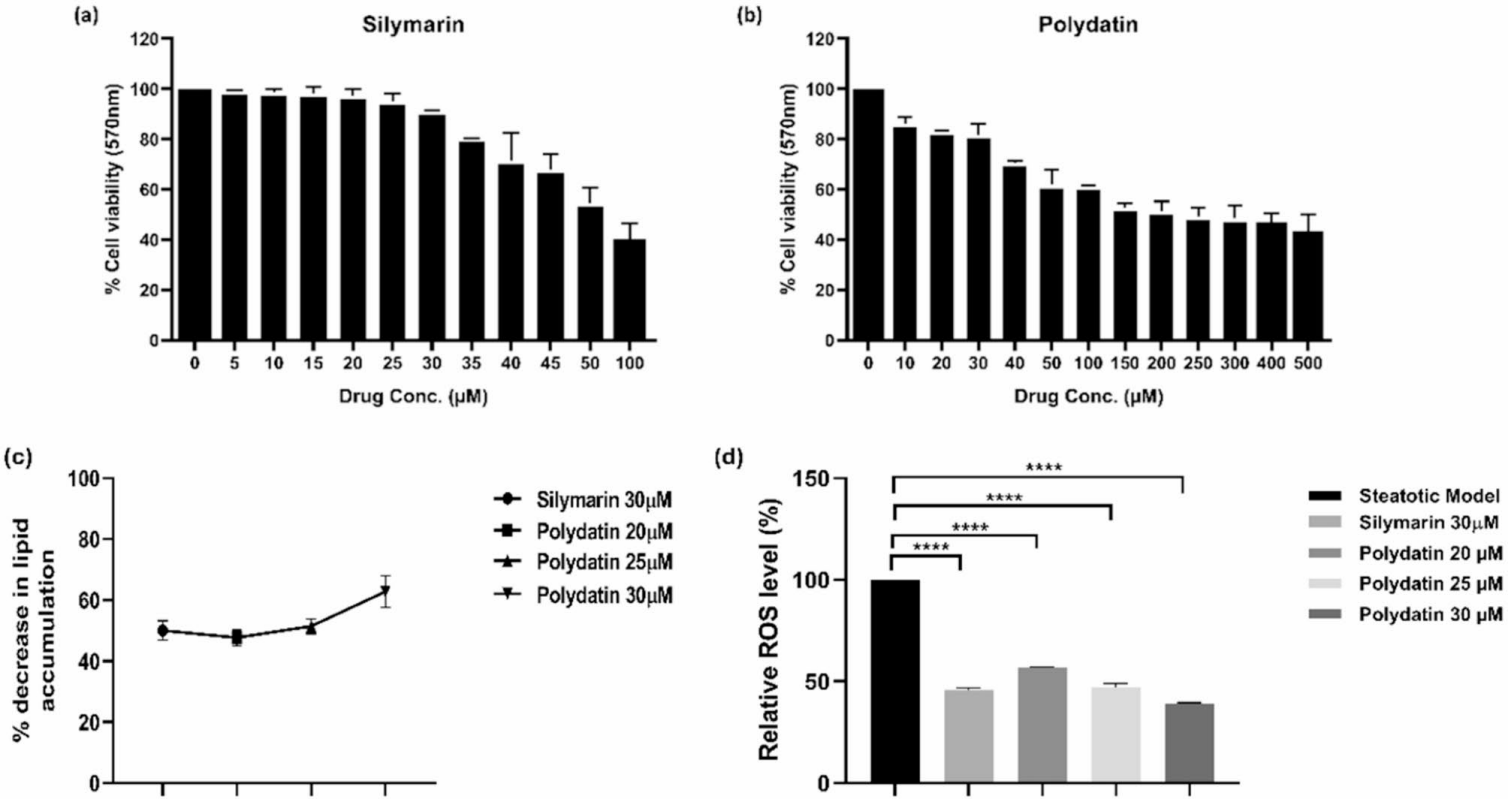
Inhibitory effects of eprosartan on lipid accumulation in in vitro model of NAFLD. (**a**) and (**b**) show the dose-dependent cytotoxicity of Silymarin and Polydatin respectively, on HepG2 cells. Dosage at which > 80% cell viability was recorded and were taken for further analysis, (**c**) Intracellular neutral-lipid contents in steatotic HepG2 cells after treatment with different concentrations of Polydatin and Silymarin, (**d**) Effects of Polydatin on intracellular ROS production in HepG2 cells. Values are expressed as mean ± S.D. of minimum of three experiments done in triplicate. Symbols **** and represent statistical significance at *p* ≤ 0.0001.

MTT cell viability assay was conducted to evaluate the HepG2 cell viability after the Polydatin treatment. The results show that treatment of HepG2 cells with Polydatin (10–50 µM) and for Silymarin (5–30 µM) when treated for 24 h did not show any toxicity, and upto the concentration of 30 µM, both the natural drugs Polydatin and Silymarin maintained more than 80% cell viability (Fig. 2a,b). Further increasing the concentration of Polydatin to 500 µM and Silymarin to 100 µM resulted in a dose-dependent cytotoxic effect. Therefore, the concentrations at which Polydatin and Silymarin showed ≥ 80% cell viability were selected for further studies^32^.

### ORO staining of neutral lipids

To investigate the mechanism of Polydatin to modulate lipid accumulation steatotic hepatocytes were exposed to OA in the presence or absence of various concentrations of (20–30 µM) Polydatin and compared with Silymarin (30 µM) (Fig. 2c). Polydatin treatment decreased neutral lipid accumulation by 62.82%, while the decrease was 50.05% following Silymarin treatment.

### Detection of intracellular ROS levels

The level of intracellular ROS was measured using the DCFDA assay. As shown in Fig. 2d, the steatotic untreated cells exhibited higher levels of ROS production, while different concentrations of Polydatin treated for 24 h significantly inhibited the rise of intracellular ROS levels (*p* < 0.0001). The 30 µM dose of Polydatin decreased ROS levels by 60.94%, while Silymarin (30 µM) treatment reduced ROS levels by 54.33%.

### Lipid peroxidation (MDA) and hepatotoxicity marker (ALT) levels

**Fig. 3:**
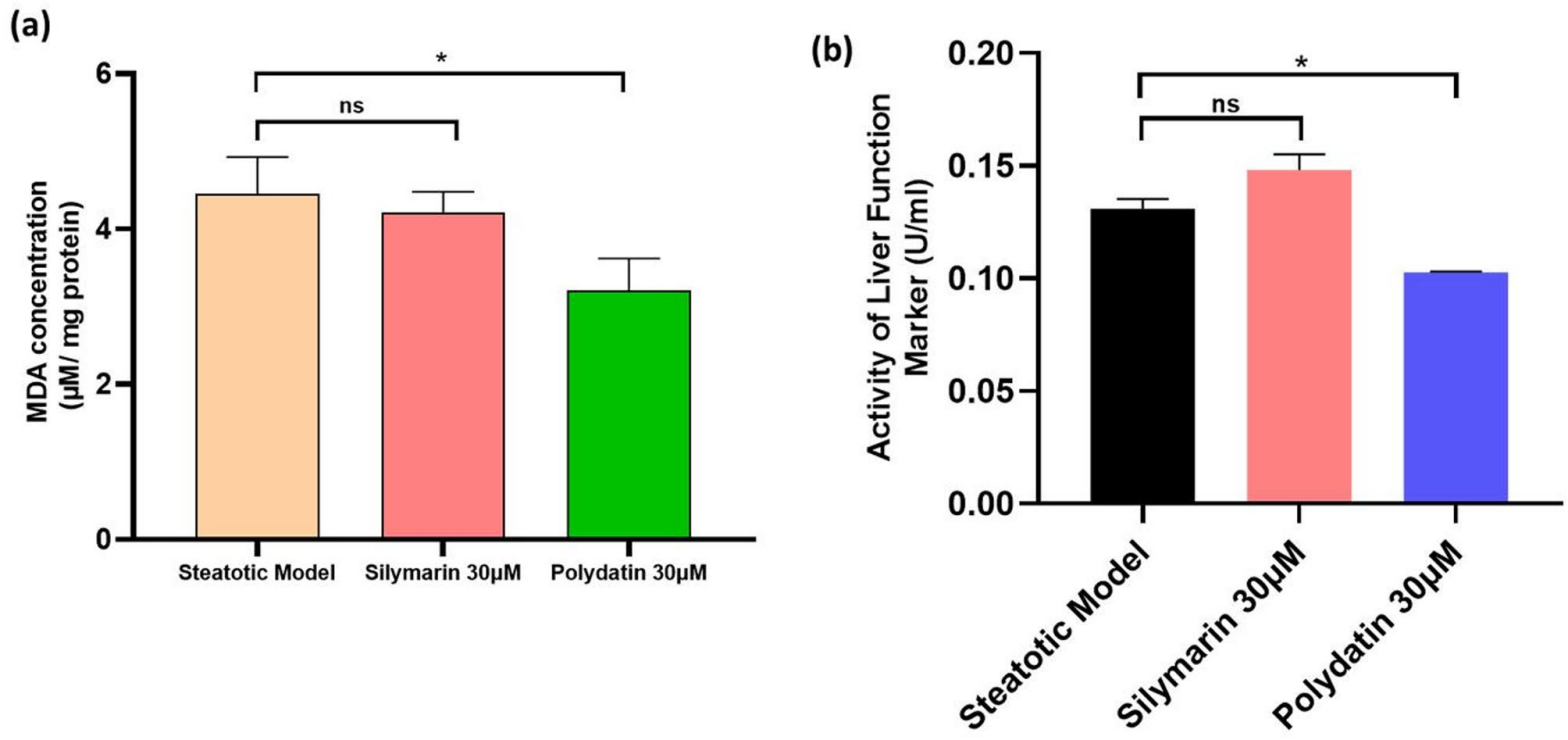
Effect of polydatin on lipotoxic damage in steatotic cells. (**a**) The oxidative stress (lipids peroxidation) levels in steatotic HepG2 cells versus Silymarin (30 µM)- and Polydatin (30 µM)-treated groups, (**b**) effects of Polydatin treatment on cell lysate ALT level. Values denote mean ± SD of three experiments done in triplicate. The Polydatin treated groups were compared to the steatotic control group, showing a statistically significant reduction in oxidative stress (*p* ≤ 0.05).

As shown in Fig. 3a, OA exposure markedly increased cellular MDA levels in the steatotic HepG2 cells. Polydatin significantly inhibited the increase of MDA levels in a dose-dependent manner, wherein 30 µM Polydatin reduced MDA concentrations by 28%. The 30 µM dose of Silymarin could reduce MDA levels by 5.47% only. The ROS levels (by DCFDA assay) results and MDA levels taken together indicate that Polydatin lowers hepatic oxidative stress levels, thus protecting the liver cells from lipotoxic damage. ALT activity, as a marker of liver damage^33^, was elevated in steatotic cells, which was significantly ameliorated by Polydatin treatment (*p* < 0.01). Silymarin did not show lower ALT activity in the present experimental conditions. (Fig. 3b).

### PPAR transcription activity assay

**Fig. 4:**
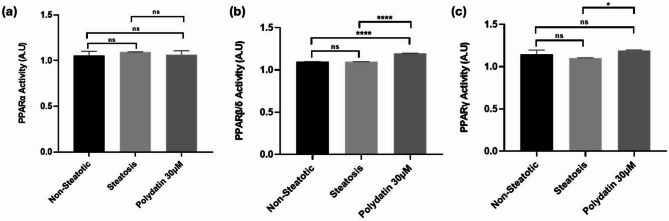
Effect of Polydatin on PPARα, β, γ DNA binding activity in nuclear extracts (**a**) PPARα activity (**b**) PPARβ/δ activity, and (**c**) PPARγ activity.

Figure 4 shows pan-PPAR(α,β,γ) activity assay analysis using a PPARα,β/δ,γ binding immunoassay. The sample groups were normalized with the same amounts of total nuclear protein to minimize variation. All three experimental groups revealed pan-PPAR activity. Polydatin treatment particularly increased the activity of PPARβ/δ and PPARγ in nuclear cell lysate compared to non-steatotic and steatotic cells. Statistically significant differences in PPAR activity were observed in PPARβ/δ and PPARγ as seen in Fig. 4.

### Effects of Polydatin on the expression of genes involved in lipogenesis and lipid oxidation

PPARα plays an important role in regulating fatty acid metabolism, particularly for many enzymes responsible for fatty acid catabolism during β-oxidation. Proteins associated with lipogenesis, such as ACC1 and FASN, are known to influence cellular energy status, whereas hepatic lipid metabolism in hepatocytes is regulated by PPARα and CPT1 through fatty acid β-oxidation.

**Fig. 5:**
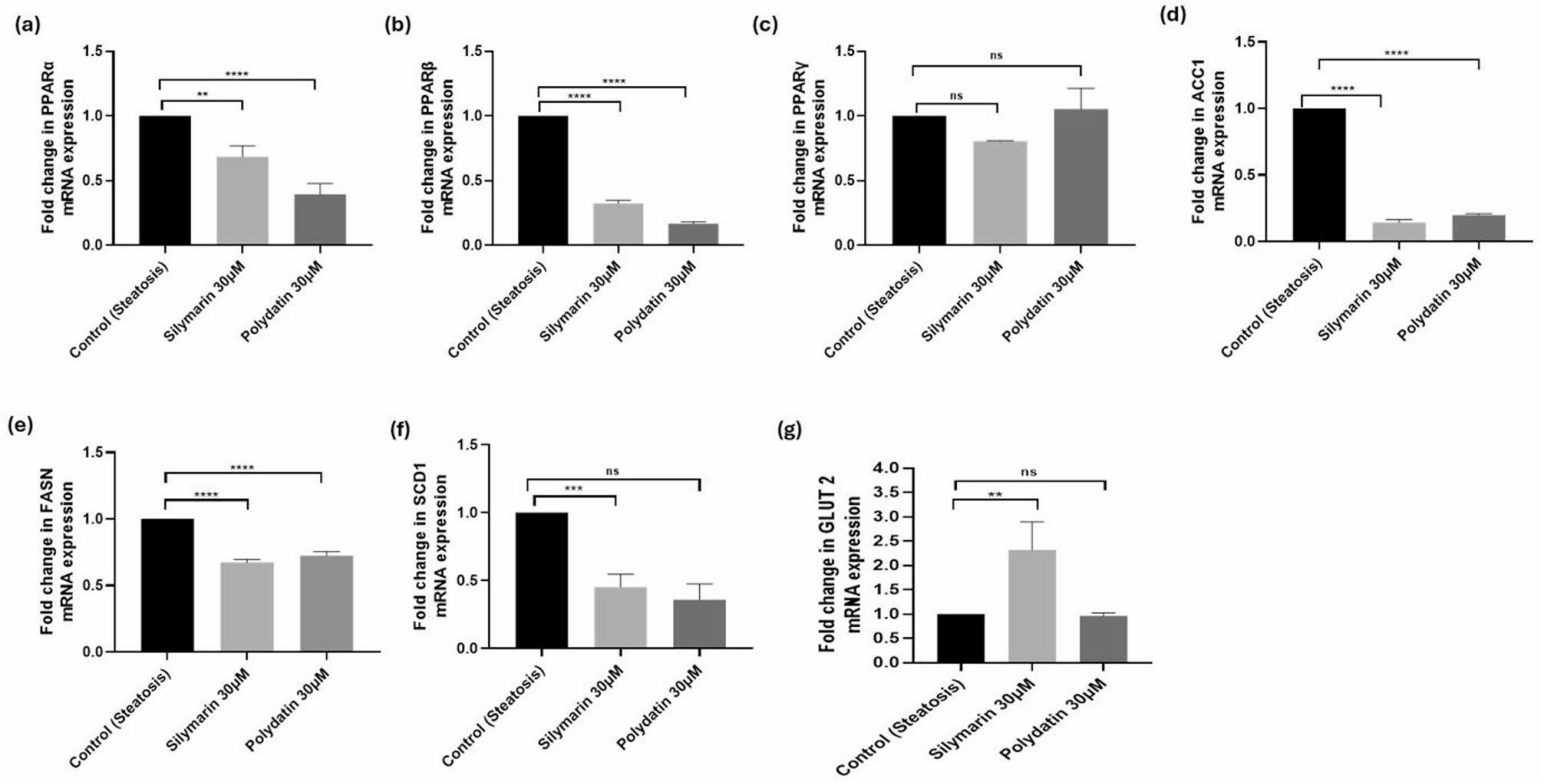
Polydatin up-regulates fatty acid metabolizing enzymes’ gene expression in HepG2 cells. Steatotic HepG2 cells were treated for 24 h with Silymarin (30µM) and Polydatin at 30µM. Figures (**a**) PPARα, (**b**) PPARβ/δ, (**c**) PPARγ, (**d**) ACC1 (**e**) FASN, (**f**) SCD1, (**g**) GLUT2. Each bar represents the mean ± SD. Statistical comparisons are with the steatotic control group. The results are expressed as fold-change calculated by the relative Ct method, using ß-actin as the internal control (housekeeping gene). Values are expressed as mean ± S.D. of a minimum of three experiments done in triplicate. Symbols **, ***, and **** represent statistical significance at *p* ≤ 0.01, 0.001, and 0.0001 respectively, and ns implies statistically not significant.

The mechanisms underlying the lipid-lowering effects of Polydatin in HepG2 cells were investigated by measuring the gene expression levels of PPARs, ACC1, FASN, SCD1 and GLUT2. Figure 5 reveals the effects of Polydatin on the gene expressions of PPARα, PPARβ, and PPARγ, wherein α and β isoforms showed decreased expression (*p* < 0.0001), and the change in γ isoform expression was non-significant. The gene expression of lipogenic markers ACC1, FASN, and SCD1 was decreased in Polydatin-treated HepG2 cells. The increase in PPAR protein expression and activity as seen in the other assays, may have impacted the transcriptional down-regulation of PPARs. These findings indicate modulation and feedback transcriptional regulation that Polydatin treatment induces on PPARs, and the downstream genes involved in lipogenesis in steatotic hepatocytes, thereby preventing fat synthesis and accumulation.

### Western blot analysis

Western blot analysis was performed to analyze the proteins involved in lipid metabolism. These include ACC1, p-ACC1, and FASN, which are related to fatty acid synthesis, as well as PPARα, which is related to fatty acid β-oxidation. Additionally, PPARβ/δ is involved in fatty acid oxidation, and PPARγ is related to energy storage by promoting adipogenesis and lipid storage. This assessment aims to evaluate the multiple effects of Polydatin on lipid metabolism. After treatment with 30 µM Polydatin in steatotic HepG2 cells, the protein expression level of lipogenic enzymes, p-ACC1 (active form), and FASN significantly decreased (*p* < 0.0001) and (*p* < 0.001), respectively. Both Silymarin and Polydatin treatments resulted in increased protein expression of lipid regulatory receptors, PPARγ (*p* < 0.0001), PPAR-α (*p* < 0.0001), and PPARβ (*p* < 0.001), which supports the direct effects of these phytochemicals on lipid metabolism.

**Fig. 6:**
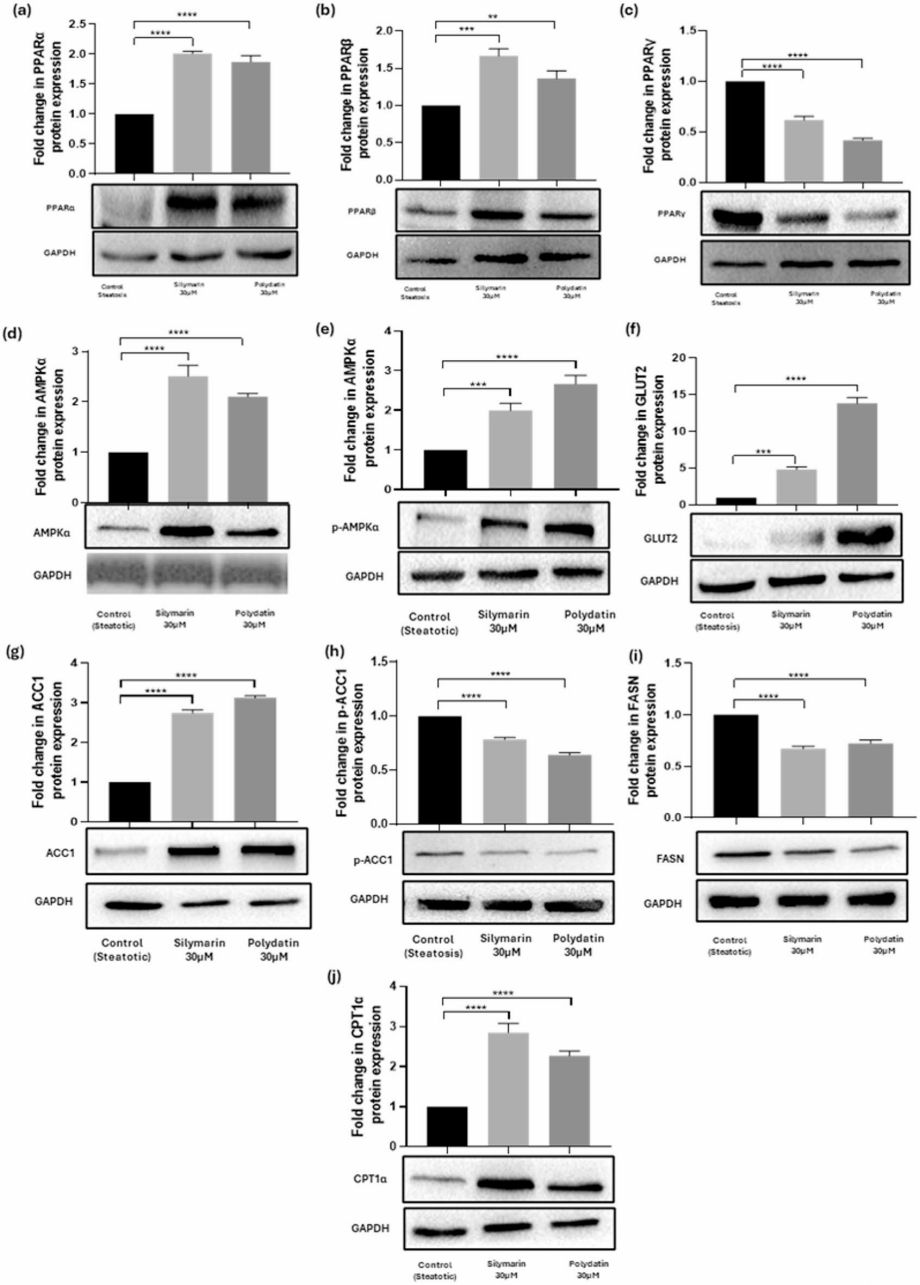
Effects of Polydatin on the hepatic protein expression levels. The lipid and energy metabolism protein expressions are shown: (**a**) PPARα, (**b**) PPARβδ, (**c**) PPAR**γ**, (**d**) AMPKα, (**e**) p-AMPKα (**f**) GLUT2 (**g**) ACC1, (**h**) p-ACC1, (**i**) FASN and (**j**) CPT1α. Values are expressed as mean ± S.D. of a minimum of three experiments done in triplicate. Symbols **, ***, and **** represent statistical significance at *p* ≤ 0.01, 0.001 and 0.0001 respectively, and ns implies statistically not significant. Original blots are presented in Supplementary Fig. 5.

In the present study, the regulatory role of Polydatin on the AMPK-mediated signalling pathway was also investigated to better understand its action on hepatic steatosis and energy metabolism. p-AMPK expression was inhibited in the steatotic HepG2 cells. However, when treated with Polydatin, the cells showed an increase in expression of p-AMPK (the active form) (Fig. 6). Previous studies have highlighted the involvement of p-AMPK activation via Ca^2+^/CaMKKβ, an enzyme that has also been reported to play a role in various physiological and pathological conditions that activate AMPK to regulate conditions such as obesity, glucose homeostasis, and cancer^34^. Studies have indicated that activation of AMPK can inhibit hepatic lipogenesis and gluconeogenesis, leading to improvement in lipid accumulation and insulin resistance^35^. Compared to the steatotic control, treatments with 30 µM Silymarin and 30 µM Polydatin significantly increased p-AMPK (*p* < 0.0001).

Consequently, activation of AMPK led to inhibition of ACC1 (that is, decreased p-ACC1 levels), which resulted in a downregulation of fatty acid synthesis in hepatocytes, leading to a decrease in triglycerides storage in these cells (revealed by ORO-staining). Further correlations of Polydatin’s action on fat accumulation were made by analyzing PPARα expression, a key protein mediating fatty acid β-oxidation in steatotic HepG2 cells. Western blotting showed that PPARα expression was significantly higher in Polydatin-treated cells than in steatotic control cells (Fig. 6). This upregulation of PPARα activity may have led to increased activity of carnitine palmitoyl transferase 1 A (CPT1α), a key enzyme involved in mitochondrial fatty acid oxidation. We assessed the gene and protein expression levels of GLUT2, the primary glucose transporter in hepatocytes, which plays a pivotal role in hepatic glucose uptake and homeostasis. In our OA-treated HepG2 cells, we observed a significant downregulation of both GLUT2 mRNA and protein levels, indicating impaired glucose transport capacity, which is a hallmark of insulin resistance in hepatic cells. These findings are consistent with previous reports demonstrating that reduced GLUT2 expression correlates with insulin-resistant phenotypes in NAFLD models^36^. GLUT2 levels showed an increase (13.84 fold) post-Polydatin treatment, suggesting its ability to improve insulin sensitivity. GLUT2 expression and activity are closely regulated by PPARs, particularly PPARγ, which, upon activation by specific ligands, can enhance GLUT2 transcription and influence glucose metabolism. This interaction plays a significant role in maintaining metabolic homeostasis, as PPARγ activation improves insulin sensitivity and modulates the liver’s response to glucose. These findings suggest that Polydatin reduces TG levels and lipotoxicity by promoting mitochondrial fatty acid β-oxidation while restricting lipid accumulation in hepatocytes.

### GC-MS analysis of key metabolites

GC-MS analysis was used for the identification of specific FFAs, amino acids, and other metabolites involved in lipid accumulation. In steatotic HepG2 cells, the intracellular free fatty acid (FFA) profile showed the characteristic elevated levels of saturated fatty acids such as palmitic acid (C16:0), stearic acid (C18:0), while also decreasing levels of the unsaturated fatty acid, oleic acid (C18:1) possibly due to SCD-1 enzyme downregulation. SCD1 is a lipogenic enzyme that generally desaturates stearic acid into oleic acid. As shown in Table 3, the palmitic acid and stearic acid concentrations in these steatotic cells were significantly higher than those in the treatment group.

**Table 3:** Identification of key metabolites by GC-MS analysis in steatotic NAFLD and after phytochemical treatment.

S. No.		Steatosis	Silymarin	Polydatin		Steatosis	Silymarin	Polydatin
Media								
	Retention time	Aqueous phase			Retention time	Organic phase		
1	3.46	Acetic acid (1.7)	Acetic acid (1.38)	Acetic acid (1.74)	3.46	Acetic acid (0.99)	–	Acetic acid (2.31)
2	13.65	–	Lactic acid (2.1)	Lactic acid (2.26)				
3	14.5		L-Alanine (1.91)	–				
4	25.1	–	L-Glutamic acid (1.98)	–				
5	25.49	Palmitic Acid (1.97)	Palmitic Acid (2.09)	Palmitic Acid (2.01)				
6	27.83	Stearic acid (2.02)	Stearic acid (2.08)	Stearic acid (2.1)				
Cell lysate								
	Retention time	Aqueous phase			Retention time	Organic phase		
1	3.46	Acetic acid (1.44)	Acetic acid (1.71)	Acetic acid (1.6)	2.41	Acetic acid (2.37)	–	–
2	13.64	Lactic acid (2.31)	Lactic acid (2.1)	Lactic acid (2.29)	16.3	Methyl palmitate (1.82)	Methyl palmitate (1.98)	Methyl palmitate (1.69)
3	14.49	L-Alanine (1.86)	L-Alanine (1.86)	–	17.99	Methyl oleate (1.69)	Methyl oleate (1.61)	Methyl oleate (1.63)
4	14.66	Glycerol (1.97)	Glycerol (1.98)	Glycerol (1.9)	18.22	Methyl stearate (2.53)	Methyl stearate (2.23)	Methyl stearate (2.16)
5	14.96	Glycine (1.84)	Glycine (1.91)	–				
6	20.43	Phosphoric acid (2.01)	Phosphoric acid (2.01)	Phosphoric acid (2)				
7	23.6	L-Aspartic acid (1.91)	–	–				
8	24.64	–	Hypoxanthine (2.05)	Hypoxanthine (2.08)				
9	25.1	L-Glutamic acid (1.99)	L-Glutamic acid (1.92)	L-Glutamic acid (2)				
10	27.83	Stearic acid (2.01)	Stearic acid (1.94)	Stearic acid (2.06)				
11	28.13	L-Pyroglutamic acid (2)	L-Pyroglutamic acid (2.01)	–				

The table presents the GC/MS analysis of important metabolites identified in both the cell-lysate and the cell-supernatant (media) samples. These analyses have been carried out both in the organic and aqueous phases. Values in brackets indicate A/H ratio.

Previously reported pathway analysis revealed significant dysregulation in several amino acid metabolic pathways associated with NAFLD, including that of tyrosine, tryptophan, branched-chain amino acids, glycine, and serine. Glycine is implicated in NAFLD treatment^37^ which was revealed in the present silymarin-treated cells, but not in the polydatin-treated cells. Decreased glutamate levels have been closely linked to the pathogenesis of NAFLD^38^. The low levels of glutamate in the untreated steatotic cells were improved in the Polydatin-treated cells in the present analysis.

Taken together, the GC-MS analysis demonstrated that Polydatin reduced all the identified saturated FFAs, including C16:0 and C18:0, and modulated other metabolites. These changes indicated that Polydatin plays a protective role in lipid metabolism in steatotic HepG2 cells and thus attenuated lipid accumulation in NAFLD.

## Discussion

Polydatin is a stilbenoid polyphenol naturally occurring in various plant sources. It belongs to the class of natural polyphenols that exhibit diverse biological functions, including antioxidant, anti-inflammatory, and metabolic regulatory effects^39^. These properties have led to its investigation in multiple models of metabolic and inflammatory diseases, making it a compound of growing interest in nutraceutical and pharmaceutical research^40^. The most common plants used for its extraction belong to the families *Vitaceae*,* Liliaceae*, and *Leguminosae*^41^. They have long been used to treat inflammation, infections, jaundice, skin burns, and hyperlipidemia^42^. This historical use supports its therapeutic relevance in liver-related disorders such as NAFLD, where inflammation and lipid dysregulation are key pathological features. Preclinical studies have demonstrated that Polydatin exhibits hepatoprotective effects in various liver injury models, supporting its traditional applications^43^. One of the most significant industrial sources for isolating Polydatin is the invasive plant species from East Asia, *Reynoutria japonica*^44^. Polydatin is a glycoside of resveratrol with enhanced antioxidant and anti-inflammatory activities as well as bioavailability compared to the parent compound resveratrol^45,46^. The addition of a glucose moiety in Polydatin increases its water solubility and stability, improving intestinal absorption and systemic bioavailability^40^. Moreover, Polydatin retains many of the therapeutic benefits of resveratrol but overcomes some of its pharmacokinetic limitations, making it more effective for clinical applications^24^. This compound occurs in nature in four major forms: trans-Polydatin, trans-resveratrol, cis-Polydatin, and cis-resveratrol^41^. Among these, the trans isomers are generally considered more biologically active due to their structural stability and binding affinity to cellular targets^47^. The specific configuration of these compounds also affects their interaction with nuclear receptors such as PPARs, which are central to metabolic regulation.

The initial high-throughput virtual screening (HTVS) led to the identification of top-scoring ligands for the three PPAR isoforms (α, β/δ, γ). These isoforms are known to regulate genes involved in fatty acid oxidation, lipogenesis, glucose metabolism, and inflammation, making them highly relevant in NAFLD pathology^48^. The subsequent steps—SP and XP mode docking—refined these results, leading to the selection of the top ligands for each isoform. After comparing the results, we identified the compounds that showed a balanced and optimal pan-PPAR agonist potential. Such pan-agonists are particularly valuable for metabolic diseases where multi-pathway modulation is required.

From the 53 pan-PPAR agonists identified in the present analysis, glucuronide compounds were excluded due to their reported possibility of poor cellular membrane permeability and high hydrophilicity, as they require active efflux rather than passive diffusion^31^. This decision is grounded in pharmacokinetics, as glucuronides tend to be excreted rapidly and often lack the ability to penetrate nuclear targets like PPARs efficiently^49^. The ligand demethyloleuropein, although showed the highest binding scores, displayed significant variation in binding energies (MMGBSA) across the PPAR isoforms. This inconsistency in binding affinity suggested that a more stable ligand might be preferable for further validation, which led us to shortlist dihydro-piceid. The next top-ranked ligand that is not a glucuronide was found to be luteolin glucoside. There are some recent reports that indicate its anti-NAFLD efficacy^50^. The active form of dihydro-piceid, trans-piceid (Polydatin), was then selected for experimental validations. Overall, binding protein-ligand complex stability, bioavailability, and consistent binding scores across multiple PPAR isoform targets were the selection criteria. This comprehensive computational screening approach ensures a rational and translational selection of therapeutic leads.

Polydatin has gained much attention for its biological properties, including potential health benefits and therapeutic effects. It has been investigated in a range of disorders, including cardiovascular diseases, diabetes, and liver injury, due to its pleiotropic effects^47^. The therapeutic and protective effects of Polydatin are mostly based on its anti-inflammatory, antioxidant, and anti-apoptotic properties^41^. These effects are crucial in managing NAFLD progression, where oxidative stress and inflammation play central roles^25^. In addition, Polydatin also exerts anti-inflammatory effects in mature adipocytes, which may involve reduced expression of MCP-1 and TNF-α, implicating a potential anti-obesity effect^51^. Its favorable Glide scores and balanced energy profile across all three PPAR isoforms (Supplementary Fig. 1) suggest strong and stable interactions with the targets, supporting its potential as a pan-PPAR agonist.

ADMET predictions for Polydatin and other top ligands showed that all compounds had acceptable scores for drug-likeness based on TPSA and LogP, where a TPSA below 140 Å² is generally associated with favorable oral bioavailability, and Polydatin met this criterion, indicating its potential to permeate biological membranes^52^ effectively. Additionally, its LogP value fell within the optimal range of 1–3, suggesting a balanced hydrophilic-lipophilic profile that supports both aqueous solubility and membrane permeability, essential for a drug-like compound^53^. Polydatin, in particular, demonstrated a promising pharmacokinetic profile with high predicted Caco-2 permeability, indicating strong potential for intestinal absorption, and a favorable blood-brain barrier (BBB) permeability score, which, while not critical for NAFLD, suggests broader therapeutic versatility, including potential benefits in neuroinflammatory or metabolic disorders with CNS involvement^24,54^. Polydatin’s profile suggests it may effectively cross biological membranes and achieve therapeutic levels, aligning with findings from similar pharmacokinetic studies^53,55^.

The 200 ns molecular dynamics simulations provided valuable insights into the stability and interactions of Polydatin with the PPAR isoforms, supporting its predicted pan-PPAR agonist potential. The RMSD plots indicated that the protein-ligand complexes remained relatively stable throughout the simulation period, with deviations less than 2.0 Å, which are within acceptable ranges for biologically stable interactions. When we carried out the molecular dynamic simulations for the native, unbound form of the protein(s), the RMSD deviations were greater compared to the deviations of the ligand-bound structures. This indicates that the ligand-bound (complex) structures show more stability during the dynamic studies when compared with the unbound state of the proteins (Fig. 1ia, iia and iiia) and Supplementary Fig. 2a–c). These stable RMSD profiles suggest that Polydatin formed persistent interactions with the active sites of PPARα, PPARβ/δ, and PPARγ, which are critical for maintaining receptor activation over time^56,57^. Detailed analysis of the binding interactions revealed key contacts between Polydatin and conserved residues such as Leu247, Ile317, and Lys358 in PPARα, Arg228, Tyr327, Leu340 and Phe363 in PPARγ, and His323, Phe327, and Tyr473 in PPARβ/δ suggest that Polydatin binds effectively and forms stable complexes with the receptor’s active sites. These interactions, including H-bonds and π-π stacking, are critical for the ligand’s binding affinity and receptor activation^58^. The root mean square fluctuation (RMSF) plots indicated limited flexibility at the ligand-binding regions of the PPARs, whereas higher fluctuations were noted at the terminal loops or solvent-exposed regions. The stability of the active site residues during the simulation supports the idea that Polydatin fits well into the ligand-binding domain (LBD) and induces minimal perturbation to the protein’s structure, an important trait for potential therapeutic ligands^59^. Furthermore, the RMSF plot of the protein residues had fewer fluctuations in the complex structure than the native structure indicating the bound ligand restricted the mobility of the active site residues (Fig. 1ib, iib and iiib, and Supplementary Fig. 2d–f). The radius of gyration (rGyr) values, ranging between 4.5 Å and 5.4 Å across different complexes, demonstrated the compactness of the protein-ligand structures and further confirmed the structural integrity of each complex during the simulation^60^. The consistent rGyr and RMSD values, along with specific, stable hydrogen bond occupancies observed across the MD trajectories, collectively reinforce the robustness of Polydatin’s interaction with all three PPAR isoforms and validate its potential as a stable, pan-PPAR modulator (Supplementary Fig. 3).

Oleic acid (OA) is widely used to induce steatosis in HepG2 cells as an in vitro model for NAFLD. At lower concentrations, particularly up to 0.75 mM, OA effectively promotes intracellular lipid accumulation without significantly affecting cell viability. Multiple studies have reported that cell viability remains above 80% at these concentrations, indicating minimal cytotoxicity. For instance, Wang et al. (2019) demonstrated that OA concentrations below 0.75 mM did not reduce HepG2 cell viability, while higher concentrations (≥ 1.0 mM) began to exhibit cytotoxic effects^61^. Similarly, other reports have confirmed that OA concentrations below 1 mM are well-tolerated by HepG2 cells over 24–48 h of treatment^62^. Thus, OA at sub-millimolar concentrations is considered safe and effective for establishing an in vitro NAFLD model without compromising cellular viability. The MTT assay demonstrated that Polydatin was well-tolerated by HepG2 cells at concentrations ranging from 10 µM to 50 µM, maintaining over 80% cell viability at 30 µM, which aligns with previous studies indicating its safety profile in hepatic cell lines^63^ (Fig. 2a,b). However, a demonstrable cytotoxic effect was observed at 500 µM, consistent with prior reports that polyphenolic compounds can exhibit mild cytotoxicity at very high concentrations due to pro-oxidant effects^41^. Therefore, 30 µM was chosen as the optimal concentration for further studies to balance efficacy and safety. To evaluate Polydatin’s impact on steatosis, Oil Red O (ORO) staining was employed, which revealed a marked reduction in intracellular lipid accumulation in oleic acid (OA)-induced HepG2 cells following treatment^64^ (Fig. 2c). This result suggests that Polydatin effectively counteracts OA-induced lipid overload and supports its lipid-lowering potential^65^. These findings are consistent with previous evidence where polyphenolic compounds such as Rutin, Caffeic acid, and Ferulic acid have demonstrated similar protective effects against hepatic steatosis by reducing lipid droplet formation and modulating lipid metabolism^66,67^.

The DCFDA assay indicated that OA-induced HepG2 cells exhibited elevated ROS levels, reflecting oxidative stress associated with lipid accumulation. Polydatin treatment significantly reduced ROS levels in treated cells (Fig. 2d), indicating its potent antioxidant capability, which is a known mechanism for reducing hepatocellular damage in steatotic liver models^68^. Polydatin’s ability to lower ROS and MDA levels (Figs. 2d and 3a) supports its role as an antioxidant, potentially reducing oxidative damage in hepatic cells. Furthermore, the pan-PPAR activity assay showed that Polydatin increased the activity of PPARα, PPARβ/δ, and PPARγ in nuclear cell lysates of HepG2 cells (Fig. 4). PPARs are crucial regulators of lipid metabolism and inflammation. The significant increase in PPARβ/δ and PPARγ activity, while PPARα activation was comparable to the standard reference compound Silymarin, indicating that Polydatin may function as a broad-spectrum PPAR agonist^69^. Given the central role of PPARs in lipid homeostasis, insulin sensitivity, and inflammation, these findings reinforce Polydatin’s potential as a multifaceted modulator in the context of NAFLD^70^.

Polydatin treatment led to a marked increase in the mRNA expression of PPARα, PPARβ, and PPARγ while reducing the expression of lipogenic markers ACC1 and FASN, indicating that Polydatin promotes a transcriptional shift toward enhanced lipid catabolism and suppressed lipogenesis (Fig. 5). This transcriptional modulation suggests that Polydatin effectively reprograms hepatic lipid metabolism, thereby offering protective effects against steatosis^70^. Activation of PPARα is known to stimulate β-oxidation of fatty acids, while PPARγ and PPARβ/δ are involved in modulating lipid uptake, adipogenesis, and inflammation, which collectively support the therapeutic relevance of a pan-PPAR agonist like Polydatin in NAFLD management^71^.

Western blot analysis further confirmed that Polydatin significantly increased protein levels of PPARα, PPARβ/δ, and PPARγ, while concurrently reducing levels of phosphorylated ACC1 (p-ACC1) and FASN proteins, reinforcing its dual role in promoting fatty acid oxidation and inhibiting fatty acid synthesis^72^ (Fig. 6). This concordance between transcript and protein expression levels suggests that the effects of Polydatin are both transcriptionally and translationally regulated, ensuring sustained biological impact. The inhibition of p-ACC1, a downstream target of AMPK, indicates suppression of the lipogenic pathway and a redirection of metabolic flux toward energy production, rather than storage^73^. In addition to its effects on PPAR signaling, Polydatin also activated AMP-activated protein kinase (AMPK), as evidenced by increased expression of phosphorylated AMPK (p-AMPK), a central energy sensor that regulates lipid and glucose metabolism under conditions of metabolic stress^46^ (Fig. 6). Activation of AMPK leads to the suppression of lipogenesis and enhancement of mitochondrial fatty acid oxidation, making it a critical therapeutic target in NAFLD and other metabolic diseases^74^. These results imply that the anti-steatotic effect of Polydatin is, at least in part, mediated through AMPK activation, which complements its PPAR agonistic effects by acting through a distinct yet synergistic signaling axis^35^. Thus, the upregulation of p-AMPK by Polydatin is a vital mechanism to counteract NAFLD^75^.

Furthermore, GC-MS analysis revealed that Polydatin reduced levels of saturated FFAs, including palmitic acid, stearic acid, and unsaturated fatty acid oleic acid (Table 2 and Supplementary Fig. 4). The reduction in these fatty acids suggests that Polydatin mitigates lipid accumulation by modulating fatty acid profiles in HepG2 cells. The reduction in glutamate levels following Polydatin treatment may reflect its positive impact on metabolic alterations associated with NAFLD. By exerting anti-inflammatory effects, enhancing insulin sensitivity, and reducing oxidative stress, Polydatin helps restore normal liver function and metabolic balance. Elevated glutamate levels in NAFLD often indicate disrupted amino acid metabolism and increased oxidative stress, so lowering these levels suggests that Polydatin is effectively modulating liver metabolism, improving overall hepatic health, and potentially mitigating the progression of NAFLD^46,76^.

Recently, the pharmacological action of Polydatin in various diseases was reviewed^39^ wherein its therapeutic targets, pharmacological mechanisms, biological activities, and health benefits were discussed. Further research into newer delivery systems and clinical trials with Polydatin have been indicated. The present outcomes point to multi-faceted anti-steatotic efficacy of Polydatin.

## Materials and methods

The computational techniques are discussed here followed by validation using experimental techniques.

### In silico database screening, molecular docking, and dynamics analysis

#### Protein preparation and grid generation

The 3D X-ray crystallographic structures of the ligand binding domain and co-activator of PPARα (PDB code 2ZNN, resolution: 2.01Å), PPARβ (PDB code 3GZ9, 2.0 Å), and PPARγ (PDB code 2ATH, resolution: 2.28 Å) were used as the starting structure for the in silico studies. The protein targets were prepared using the protein preparation wizard of Schrodinger software by removing crystallographic water molecules and adding hydrogen atoms, followed by minimization and optimization. Several different sets of fields were used to depict the shape and features of the receptor on a grid, providing increasingly more accurate scoring of the ligand poses.

### pan-PPAR targeted HTVS of natural compounds database: ligand preparation and molecular docking

Several natural product molecules databases are currently available for ligand screening, such as coconut, SNDP, ZINC natural database. In the present study, PhytoHub database was selected for high throughput screening of the molecules, considering ease of availability of the chemical information and sourcing of the molecules. The chemical structures were obtained from the PubChem database and then extracted for ligand preparation.

The virtual screening of the natural compounds was performed using Maestro software against the binding sites of PPARs (α,β/δ,γ) protein targets. The workflow involves ligand preparation using LigPrep, skipping the step since a prior LigPrep was performed for the database. Then, the receptor grid file was added, and screening-based docking was performed with default parameters, such as using Epik state penalties for docking. The docking accuracy level was set to High Throughput Virtual Screening (HTVS) for screening the natural compound database. The scaling factor was kept at the default value of 0.8, and the partial charge cut-off was set at 0.15. The OPLS_2005 force field was used during the docking process.

### Molecular mechanics/generalized born surface area (MMGBSA) analysis

The MMGBSA analysis was further performed for refining lead hit identification. The MM-GBSA method calculates binding free energies for the best hit-docked complexes using MM force fields and implicit solvation. This method is very useful in computing the relative binding affinities that each hit requires for the target protein. The method is used as a filter to shortlist the ligands for in vitro validation. The Prime module version 3.0 was used to determine the binding free energies of the docked complexes. This protocol uses MM-GBSA to find the different binding energies of the complex, ligand, and receptor. The pose viewer file was used to generate the binding energy calculations. All protein atoms were rigid, and only the ligand structure was relaxed by default. The binding energy was calculated based on the following equation. The protein-ligand complexes were ranked based on their free energy calculation.

### Molecular dynamics simulations

Molecular Dynamics (MD) simulation helps visualize the Protein–Ligand complex (PLC) action at the target’s binding site region under physiological conditions. MD was performed using Desmond module of Schrödinger developed by D.E Shaw research group (Academic license, Version 2020-1)^77^. The cubic simulation box was prepared with the Transferable Intermolecular Potential with 3 Points (TIP3P) water model in such a way that the minimum distance between the protein surface and the solvent surface is 10 Å. Complexes docked with receptors were solvated with the cubic TIP3P water model^78^. Neutralization of the solvated system was accomplished by counter ions and limiting the salt concentration in the physiological system to 0.15 M. The PLC system was designated with an OPLS AA force field^79,80^.

Two seconds of relaxation time were used for the Reversible reference system Propagator Algorithms (RESPA) integrator^81^, Nose–Hoover chain thermostat^82^and Martyna–Tobias–Klein barostat. The final production of MD simulation was performed using the equilibrated system. This MD simulation time was set to run for 200 ns with 310 K temperatures at 1.0 bar pressure with NPT (Isothermal-Isobaric ensemble was used to maintain constant temperature, constant pressure, constant number of particles) ensemble at default settings^83^ for relaxation before simulation. The MD simulation was performed with the MD simulation tool. The trajectory was written with 1000 frames during the complete MD simulation. The protein backbone frames were aligned to the backbone of the initial frame to better understand the complex’s stability during MD simulation. Finally, after loading the .out file and selecting the Root Mean Square Deviation (RMSD) and Root Mean Square Fluctuation (RMSF) in the analysis type to oblique, the simulation interaction diagram and the results were analyzed^84,85^.

### Pharmacokinetic (ADMET) analysis

Undesirable pharmacokinetics and toxicity of candidate molecules are the leading reasons for drug development failure. Absorption, distribution, metabolism, excretion, and toxicity (ADMET) of chemicals have long been recognized as critical concerns. Based on large experimental data sets, the pKCSM online server was used to evaluate the pharmacokinetics and drug-likeness profile of the titled small molecules^86^. The SMILES format of the molecules was given as input, and pKCSM generated the 2D structure files. The pkCSM predicts small-molecule pharmacokinetics using graph-based signatures (created cooperatively by Instituto Rene RachouFiocruz Minas, The University of Melbourne, and The University of Cambridge).

Several factors are examined to verify the ADMET characteristics of a small molecule or inhibitor. The bioavailability score, as well as pharmacokinetics parameters including human intestinal absorption, P-glycoprotein, BBB, and drug-likeness prediction based on Lipinski, Ghose, and Veber criteria, are crucial in judging the molecule. Based on various important characteristics such as molecular weight, LogP, number of HPA, HBD, the Lipinski, Ghose and Veber guidelines were used to assess drug-likeness in order to determine whether a molecule is likely to be bioactive. According to Lipinski’s “Rule of 5” most “drug-like” compounds have logP ≤ 5, molecular weight (MW) ≤ 500, number of hydrogen bond acceptors (nHA) ≤ 10, and number of hydrogen bond donors (nHD) ≤ 5. Bioavailability issues may arise if any chemical moiety violates more than one of these rules.

### Experimental validation of anti-steatotic (NAFLD) efficacy of Polydatin in vitro

#### Cell culture

HepG2 cells (National Centre for Cell Science -Pune, India), were cultured in Minimum Modified Medium (MEM), 10% FBS, and 1% antibiotics and incubated in a CO_2_ incubator at 37 °C. Steatosis was induced by the 1 mM oleic acid (OA) dissolved in 1% BSA and then incubated for 48 h. The steatotic cells were then treated with Polydatin for 24 h, and results were compared with a known hepatoprotectant phytochemical, Silymarin^56^.

### Cytotoxicity study

To assess cell viability of HepG2 cells in response to various concentrations of polydatin and Silymarin, we employed the 3-(4, 5-dimethylthiazol-2-yl)-2, 5-diphenyl tetrazolium bromide (MTT) assay. HepG2 cells were cultured in 96-well plates and treated with different concentrations of polydatin and Silymarin for 24 h. Following treatment, 100 µl of MTT solution (at a concentration of 0.5 mg/ml) was added to each well, and the cells were further incubated at 37 °C for 3 h. To dissolve the formazan crystals, 100 µl of dimethyl sulfoxide (DMSO) was added to each well. The absorbance of the solubilized blue formazan was then measured at 570 nm using a microplate reader. The reduction in optical density, caused by polydatin and Silymarin treatment, was used to determine cell proliferation, with untreated cells as control serving as the baseline for 100% viability. The outcome served to optimize the safe dosage for further analysis.

### Evaluation of lipid accumulation by oil red O staining

To evaluate changes in lipid accumulation, steatotic HepG2 cells (5 × 10^4^ cells) were treated with either Silymarin (30 μm) or Polydatin (20–30 μm). The cells were washed twice with PBS and then fixed in 4% paraformaldehyde for 20 min. They were subsequently stained with Oil Red O solution for 30 min at room temperature. The lipid droplets stained with ORO were observed under a microscope. To quantify the cellular lipid content, the dye was extracted with 100% isopropanol and measured spectrophotometrically at 540 nm^87^.

### Activity measurement of the enzyme marker of liver disorder

Alanine transaminase activity (ALT) was measured in the cell lysate using an activity assay kit (Cat. #700260 Cayman Chemicals, USA), in accordance with the manufacturer’s instructions. Briefly, around 5 × 10^6^ cells were seeded for the experiment, and after phytochemical treatment, the cell lysate was collected. 150 µl of substrate, 20 µl of cofactor, and 20 µl of sample were added to the cell lysate in the wells. The plate was then covered and incubated at 37 °C for 15 min. After removing the plate cover, 20 µl of ALT Initiator was quickly added to initiate the reactions in all the wells being used. The absorbance at 340 nm was immediately measured once every minute for 10 min at 37 °C^88^.

### PPAR activation assay

#### Nuclear fractionation

The nuclear fractionation was performed with some modifications of the previously reported method^89^. Briefly, cells were seeded at a density of 1.3 × 10^5^ cells cm^2^ in 6-well plates and incubated at 37 °C overnight. Cells were then exposed to phytochemicals. At the end of the culture, the cells were washed in ice-cold PBS before lysis with cytoplasmic extraction buffer (10 mM HEPES, 60 mM KCl, 1 mM EDTA, 0.0075% (v/v) NP40, 1 mM DTT, and 1 mM PMS, pH adjusted to 7.6). Cells were then incubated on ice for 3 min, followed by centrifugation at 150 × g at 4 °C for 4 min. The supernatant was removed and gently resuspended in cytoplasmic extraction buffer without NP40. Following centrifugation at 150 × g at 4 °C for 4 min, the supernatant was removed, and the pellet was resuspended in nuclear extraction buffer (20 mM Tris, 420 mM NaCl, 1.5 mM MgCl_2_, 0.2 mM EDTA, 1 mM PMSF, pH adjusted to 8). After 10 min incubation on ice, the extract was centrifuged at 15,000 g speed at 4 °C for 10 min. The supernatant (containing the nuclear fraction) was then transferred to a fresh tube^89^.

#### PPAR activity assay

PPAR activity was determined using a PPAR (alpha, delta, gamma) Transcription Factor Assay Kit (Cayman, Michigan, USA). In brief, nuclear protein extracts (prepared as mentioned above) were loaded into wells coated with PPAR-α, PPAR-β/δ, or PPAR-γ consensus DNA, and the plates were stored at 4 °C overnight. The plate was then washed 5 times with a wash buffer to remove unbound reagents. Primary antibody was added, and the plate was incubated at room temperature for 60 min. After another wash period, goat anti-rabbit-HRP conjugate secondary antibody was added and the plate was stored at room temperature for an hour. Thereafter, the transcription factor developing solution was added for 15–45 min, followed by the addition of a stop solution. The plate was then read at 450 nm using a microplate spectrophotometer^90^.

#### Fluorimetric assay of reactive oxygen species

To evaluate reactive oxygen species (ROS) levels within cells, the 2′,7′-dichlorofluorescein diacetate (DCFDA) assay was performed. The HepG2 cells were seeded onto 96-well plates at a density of 10,000 cells per well and cultured in MEM medium till the cells became confluent. The HepG2 cells were treated with oleic acid as indicated previously, to induce steatosis. Subsequently, the steatotic cells were washed with 100 µL of PBS per well and incubated with 30 µM Silymarin and the different concentrations of Polydatin (20–30 µM). Then, after 24 h of incubation, 10 µM DCFDA was added to each well and incubated at 37 °C for 60 min, allowing the probe to penetrate the cells. The fluorescence readings were taken after 60 min. Fluorescence measurements were conducted using a luminescence plate reader (Thermo Fisher, USA), with excitation set at 485+/-10 nm and emission read at 520±25 nm^91^.

#### Lipid peroxidation assay (marker of oxidative stress)

To evaluate the effects of Polydatin and Silymarin on oxidative stress, levels of malondialdehyde (MDA), a stable by-product of lipid peroxidation was measured using the thiobarbituric acid reactive substances (TBARS) assay. The control group included steatotic HepG2 cells that did not receive Polydatin treatment. In the treatment groups, steatotic HepG2 cells were exposed to varying concentrations of Polydatin (20–30 µM) and Silymarin (30 µM) for 24 h. Following treatment, cells were lysed using a lysis buffer, homogenized, and centrifuged at 13,000×g for 15 min at 4 °C to prepare for the lipid peroxidation assay. The supernatant was collected for further testing. Protein concentrations in the cell lysates were standardized to 1 mg/ml and then mixed with 250 µl of 10% trichloroacetic acid (TCA). The mixture was subsequently combined with 375 µl of a 1% (w/v) thiobarbituric acid (TBA) solution under acidic conditions. This solution was heated in a boiling water bath for 15 min, leading to the formation of a pink-colored adduct. The absorbance of this adduct was measured at 530 nm using a Multiskan FC spectrophotometer (Thermo Scientific, USA). The values were expressed as micromolar concentrations (µM) of malondialdehyde per milligram (mg) of protein^56^.

#### Gene expression analysis

HepG2 cells treated with 30 µM polydatin and 30 µM of Silymarin for 24 h were subjected to RNA extraction using Trizol reagent (BioRad). The extracted total RNA was converted into cDNA using the Reverse Transcription System Kit (Thermo Scientific, USA). Quantitative real-time PCR (qRT-PCR) was performed using SYBR Green master mix (BioRad, US) as per the manufacturer’s protocol. The relative mRNA expression levels of genes such as PPARγ, PPARα, PPARβ/δ, ACC1, SCD1, FASN, and GLUT2 were normalized to β-actin and quantified using the 2^−ΔΔCt^ method. Each experiment was carried out in triplicate.

#### Western blot analysis

HepG2 cells were treated with Polydatin at concentrations of 30 µM and 30 µM of Silymarin for 24 h, then lysed using RIPA lysis buffer. The supernatant containing total cellular protein extracts was collected after centrifugation at 12,000 g for 5 min at 4 °C. Protein samples were separated by 10% SDS-PAGE and subsequently transferred to a polyvinylidene difluoride (PVDF) membrane. The membranes were blocked with 5% skim milk in TBST at room temperature for 1 h. Following blocking, the membranes were incubated overnight at 4 °C with primary antibodies: anti-rabbit PPARα (1:1000), anti-rabbit PPARβ/δ (1:1000), anti-rabbit PPARγ (1:1000), anti-rabbit p-ACC1 (1:1000), anti-rabbit FASN (1:1000), anti-rabbit GLUT2 (1:1000), and anti-rabbit GAPDH (1:3000). After three washes with TBST, the membranes were incubated with appropriate HRP-linked secondary antibodies. Protein bands were visualized using the Clarity Western ECL Substrate (BioRad, USA) according to the manufacturer’s protocol.

### GC-MS analysis of key metabolites

After treatments, the culture medium was collected, and the cells were washed twice with 0.9% NaCl. The metabolic activity of the cells was quenched by 1.5 ml of cold methanol, and the cells were placed on ice and was processed as described by García et al.^92^. The dried samples were resuspended in 100μl of hexane and transferred to GC vials for GC-MS analysis (Shimadzu).

For the aqueous samples, the derivatization was done by using methoxylamine hydrochloride, (MOX), N-methyl-N-(tert-butyl-dimethylsilyl)-trifluoroacetamide (MTBSTFA) and tert-butyldimethylchlorosilane and incubated at 60 C for 30 mins. After incubation, the samples were transferred to GC vials for GC-MS analysis.

### Statistical analysis

The in vitro experiments were performed as at least two to three independent experiments, each in triplicate sets. Statistical analysis was performed (GraphPad Prism) using one-way ANOVA to compare the experimental groups, and the statistically significant differences in mean values were expressed as: *P* ≤ 0.05 (*), *P* ≤ 0.01 (**), *P* ≤ 0.001 (***) and *P* ≤ 0.0001 (****).

## Conclusion

The HTVS and in silico analysis of natural product database generated about 53 top-scoring ligands with pan-PPAR activity. Using certain selection criteria (evaluating PLC stability through molecular dynamics simulation and pharmacokinetic profiling using pkCSM), Polydatin was chosen for biochemical testing in a steatotic model in vitro. Polydatin afforded protection against hepatic steatosis, by inhibiting hepatic lipid synthesis and accumulation, and promoting lipid oxidation and utilization (energy metabolism), and reducing oxidative stress. Detailed studies are required to further investigate the molecular mechanisms of Polydatin and its in vivo pharmacokinetics. The present outcomes strongly indicate the potential for Polydatin as a nutraceutical and for adjunct therapy in NAFLD and may also be extended to other MetS conditions. The other top-ligands can also be experimentally evaluated and tested with suitable delivery systems.

## Electronic supplementary material

Below is the link to the electronic supplementary material.

Supplementary Material 1

## Data Availability

Data described in the manuscript will be made available upon request pending application and approval from the corresponding author.

## Abbreviations

ALT: Alanine transaminase activity
BSA: Bovine serum albumin
DCFDA: 2′,7′-Dichlorofluorescein diacetate
DMSO: Dimethyl sulfoxide
PPAR: Peroxisome proliferator-activated receptors
FFA: Free fatty acid
HTVS: High throughput virtual screening
MEM: Minimum essential medium
MetS: Metabolic syndrome
MD: Molecular dynamics
MDA: Malondialdehyde
MTT: 3-(4, 5-dimethylthiazol-2-yl)-2, 5-diphenyl tetrazolium bromide
NAFLD: Non-alcoholic fatty liver disease
NASH: Non-alcoholic steatohepatitis
OA: Oleic acid
OPLS-2005: Optimized potentials for liquid simulations
PLC: Protein–ligand complex
PDB: Protein data bank
RESPA: Reversible reference system propagator algorithms
ROS: Reactive oxygen species
RXR: Retinoid X receptor
SP: Standard precision
SPC: Simple point-charge
TCA: Trichloroacetic acid
TBARS: Thiobarbituric acid reactive substances
XP: Extra precision
