# Supplementary material for: In silico and in vitro investigations reveal pan-PPAR agonist activity and anti-NAFLD efficacy of polydatin by modulating hepatic lipid-energy metabolism
Source: Sci Rep. 2025 Jul 24;15:26995. doi: 10.1038/s41598-025-12357-0 (PMC12290051; doi:10.1038/s41598-025-12357-0)
Supplement: Supplementary file 1 — Supplementary Material 1 [file 41598_2025_12357_MOESM1_ESM.docx]

***In silico and in vitro investigations reveal pan-PPAR agonist activity and anti-NAFLD efficacy of Polydatin by modulating hepatic lipid-energy metabolism***

**Sumit Kumar Mandal^a^, Mohammed Muzaffar-Ur-Rehman^b^, Sonakshi Puri^a^, Pankaj Kumar Sharma^a^, Sankaranarayanan Murugesan^b^, P R Deepa^a*^**

^a^Biochemistry and Enzyme Biotechnology, Department of Biological Sciences, Birla Institute of Technology and Science Pilani, Pilani Campus, Pilani-333 031, Rajasthan, India.

^b^Medicinal Chemistry Research Laboratory, Department of Pharmacy, Birla Institute of Technology and Science Pilani, Pilani Campus, Pilani-333 031, Rajasthan, India.

*Corresponding author

Corresponding author: Prof. P.R. Deepa

Address: Dept. of Biological Sciences, Birla Institute of Technology and Science Pilani, Pilani Campus, Pilani-333 031 (Rajasthan), INDIA

Tel: +91 1596 255881, E-mail: [deepa@pilani.bits-pilani.ac.in](mailto:deepa@pilani.bits-pilani.ac.in)

**Supplementary Table 1:** Docking scores of the top ligands at the active sites of PPARα, β/δ,γ

|  |  | **2ZNN (PPARα)** | **3GZ9 (PPARβ/δ)** | **2ATH (PPARγ)** |
| --- | --- | --- | --- | --- |
| 1 | Demethyloleuropein | -12.209 | -13.126 | -14.516 |
| 2 | Eriodictyol-7-glucuronide | -13.046 | -12.049 | -12.375 |
| 3 | 5-(3'-hydroxyphenyl)-gamma-valerolactone-4'-O-beta-D-glucuronide | -11.424 | -12.379 | -12.023 |
| 4 | cis-Resveratrol-3-O-glucuronide | -12.112 | -11.013 | -11.942 |
| 5 | Protocatechuic acid-3-O-glucuronide | -9.976 | -10.762 | -11.877 |
| 6 | 6-Hydroxyluteolin 7-O-rhamnoside | -12.186 | -12.662 | -11.819 |
| 7 | (-)-Epicatechin 4'-O-glucuronide | -10.342 | -11.031 | -11.759 |
| 8 | (-)-Epicatechin-5-O-glucuronide | -11.871 | -12.064 | -11.710 |
| 10 | Farnesol glucuronide | -10.555 | -11.585 | -11.604 |
| 11 | Luteolin 7-O-glucoside | -11.508 | -11.104 | -11.603 |
| 12 | 3-(3?-Hydroxyphenyl)propionic acid-glucuronide | -10.576 | -10.829 | -11.553 |
| 13 | Dihydro-resveratrol-3-O-glucuronide | -11.759 | -11.582 | -11.500 |
| 14 | 3-Phenylpropionic acid-4'-O-glucuronide | -8.032 | -10.783 | -11.475 |
| 15 | dihydro-piceid | -11.392 | -11.898 | -11.460 |
| 16 | cis-Resveratrol-4'-O-glucuronide | -11.034 | -11.849 | -11.444 |
| 17 | Isourolithin A-3-glucuronide | -10.556 | -9.287 | -11.428 |
| 18 | Perillic acid glucuronide | -8.686 | -10.543 | -11.198 |
| 19 | Scutellarin | -12.842 | -8.853 | -11.126 |
| 20 | 3-O-Caffeoylquinic acid | -10.136 | -9.661 | -11.057 |
| 21 | 5-O-Caffeoylquinic acid | -10.136 | -9.661 | -11.057 |
| 22 | Neochlorogenic acid | -10.136 | -9.661 | -11.057 |
| 23 | resveratrol-3-O-glucuronide | -11.628 | -11.258 | -11.044 |
| 24 | trans-resveratrol-5-O-glucuronide | -11.628 | -11.258 | -11.044 |
| 25 | (+)-Catechin 3-O-glucose | -10.582 | -11.940 | -11.015 |
| 26 | Benzoic acid-4-O-glucuronide | -9.369 | -10.244 | -10.951 |
| 27 | hydroxy-p-menth-8-en-7-oic acid glucuronide II | -10.566 | -11.011 | -10.939 |
| 28 | Astragalin | -9.360 | -12.713 | -10.861 |
| 29 | Kaempferol 3-O-glucoside | -9.360 | -12.713 | -10.861 |
| 30 | Resveratrol ribosyl sulfate | -10.731 | -10.890 | -10.790 |
| 31 | Phenylacetic acid-3?-sulfate-4?-glucuronide | -9.645 | -9.808 | -10.784 |
| 32 | Dopa-betaxanthin | -9.641 | -11.237 | -10.727 |
| 33 | Oxyresveratrol 3?-O-?-D-glucoside | -11.922 | -11.654 | -10.698 |
| 34 | 2-methyl-3-(5'-carboxy-3'-methyl-2'-pentenyl)-1,4-naphthoquinone | -9.116 | -8.413 | -10.672 |
| 35 | 5-(Phenyl)valeric acid-3?-glucuronide | -8.907 | -11.427 | -10.659 |
| 36 | Naringenin-7-glucuronide | -11.392 | -10.953 | -10.621 |
| 37 | Oxyresveratrol-2-O-?-D-glucuronosyl | -12.652 | -11.282 | -10.573 |
| 38 | Puerarin | -10.618 | -10.068 | -10.546 |
| 39 | DIBOA glucoside | -10.022 | -10.776 | -10.541 |
| 40 | Phloretin-2'-O-glucoside | -10.626 | -11.215 | -10.515 |
| 41 | hydroxy-p-menth-8-en-7-oic acid glucuronide I | -10.520 | -10.296 | -10.486 |
| 42 | Trihydroxybenzoic acid-glucuronide | -10.036 | -11.336 | -10.479 |
| 43 | Tangutorid E | -9.237 | -9.564 | -10.407 |
| 44 | Tangutorid F | -9.237 | -9.564 | -10.407 |
| 45 | 5-Carboxyresorcinol 3-O-beta-D-Glucuronide | -9.481 | -10.277 | -10.380 |
| 46 | 4'-Hydroxyphenylacetic acid-3'- glucuronide | -8.838 | -10.243 | -10.346 |
| 47 | (?)-Epicatechin-7-sulfate | -9.123 | -9.504 | -10.274 |
| 48 | Dihydroquercetin | -8.289 | -8.770 | -10.150 |
| 49 | 3-O-p-Coumaroylquinic acid | -10.595 | -10.397 | -10.133 |
| 50 | Phloretin-sulfate | -8.106 | -9.198 | -10.115 |
| 51 | 5-(Dihydroxyphenyl)-?-valerolactone-glucuronide | -9.116 | -11.832 | -10.113 |
| 52 | Protocatechuic acid 4-O-glucoside | -8.807 | -10.865 | -11.069 |
| 53 | HBOA glucoside | -9.350 | -11.634 | -10.061 |

**Supplementary Table 2:** List of genes with primer sequences. The gene name and primer sequences used in the gene expression study.

| **Gene name** | **Sense/ antisense: Sequences** |
| --- | --- |
| PPARγ | **F:** TGTCGGTTTCAGAAGTGCCTTG |
|  | **R:** TTCAGCTGGTCGATATCACTGGAG |
| PPARα | **F:** TCGAGGAAGGCACTACACCT |
|  | **R:** TCTTCCCAAAGCTCCTTCAA |
| PPARβ/δ | **F:** GCCGCCCTACAACGAGATCA |
|  | **R:** CCACCAGCAGTCCGTCTTTGT |
| ACC1 | **F:** ATGTCTGGCTTGCACCTA |
|  | **R:** CCCCAAAGCGAGTAACAAATT |
| SCD1 | **F:** CGTCGTCTTCAGACACACCA |
|  | **R:** GGTCGTGAGCTCAATTCCCA |
| FASN | **F:** GAAGGAGGGTGTGTTTGCC |
|  | **R:** GGATAGAGGTGCTGAGCC |
| GLUT2 | **F:** TTGGTGGGTGGCTTGGGGAC |
|  | **R:** ACCAGGCCTGAAATTAGCCCACA |

**
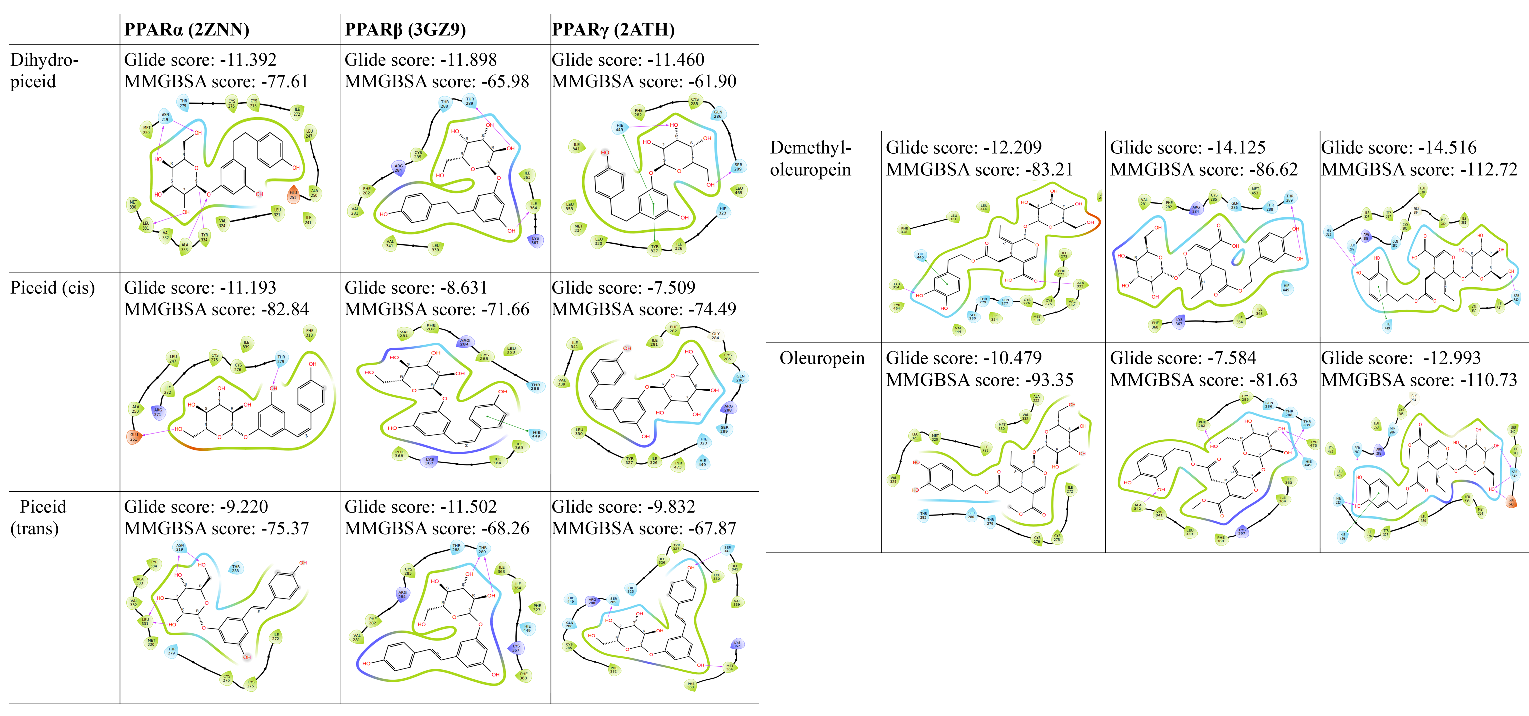
**

**Supplementary Figure 1:** Molecular docking images of the compounds with their binding free energy scores (MMGBSA).


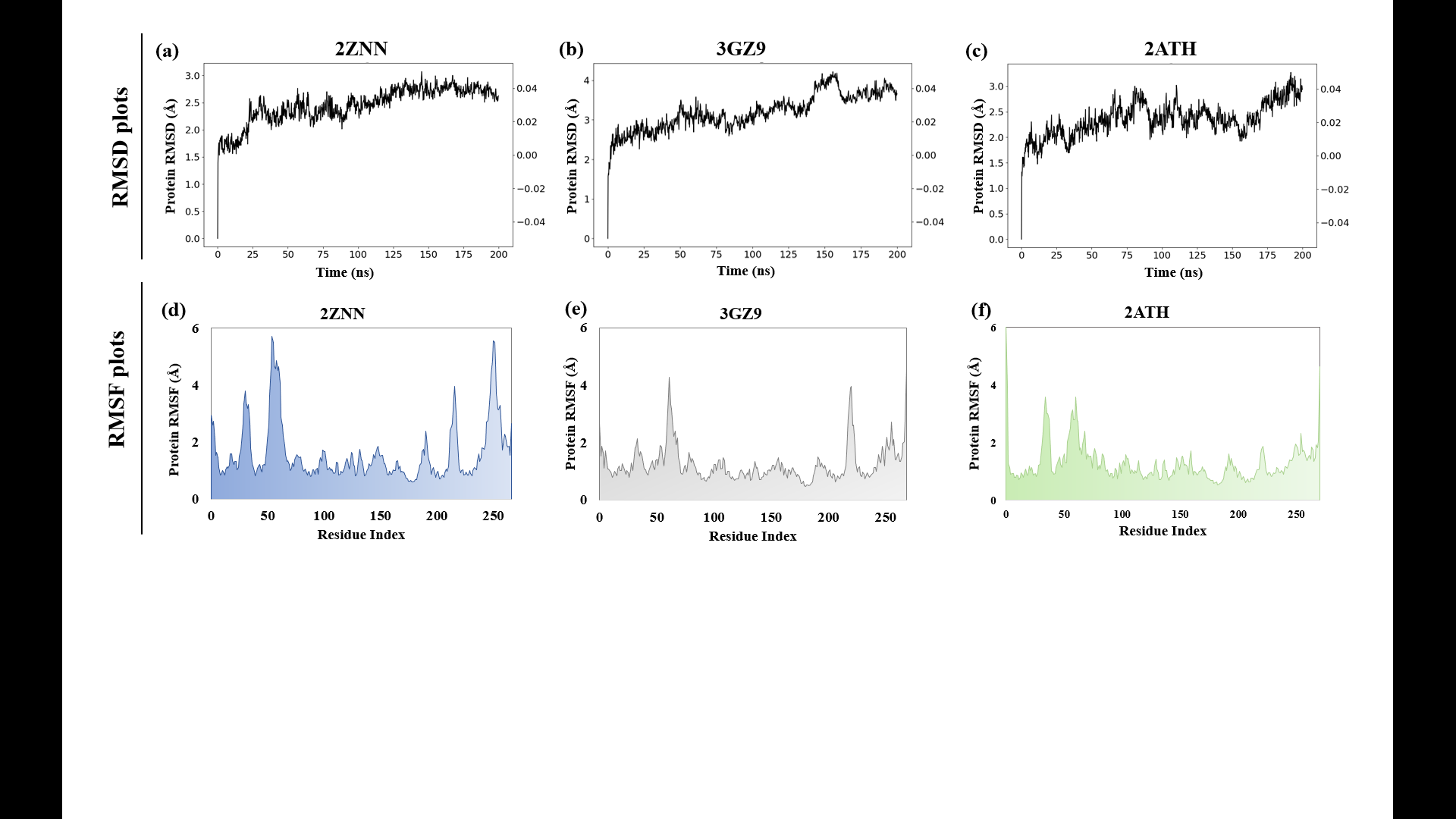


**Supplementary Figure 2:** Root mean square deviations (RMSD) and root mean square fluctuations (RMSF) of PPARα, β, and γ in the apo-form from the molecular dynamic simulations. (a-c) represents the RMSD plots, and (d-f) represents the RMSF plots for 200 ns


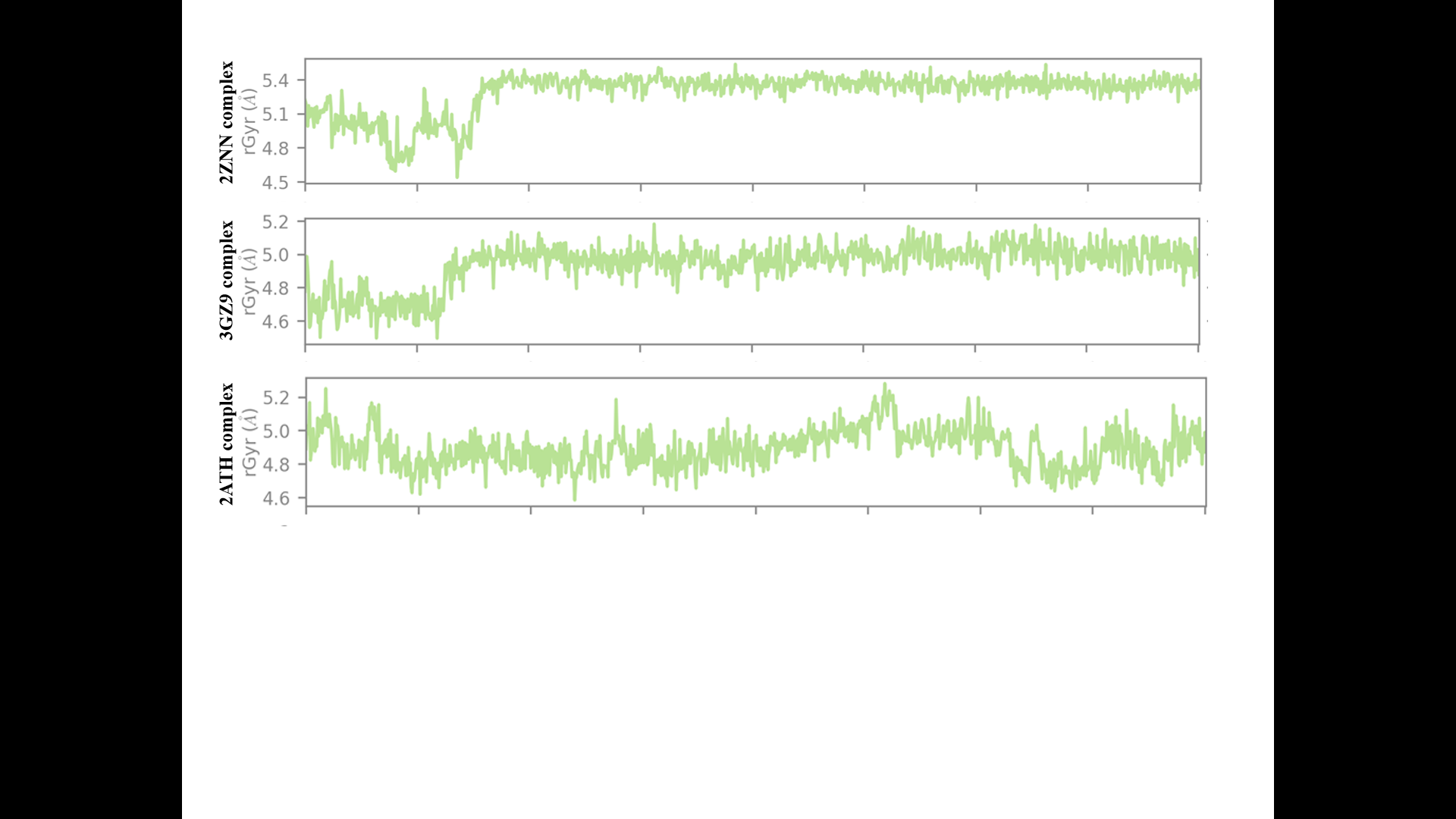


**Supplementary Figure 3:** Radius of gyration of polydatin with pan-PPAR receptors showing compactness with the protein during the molecular dynamic simulations

**
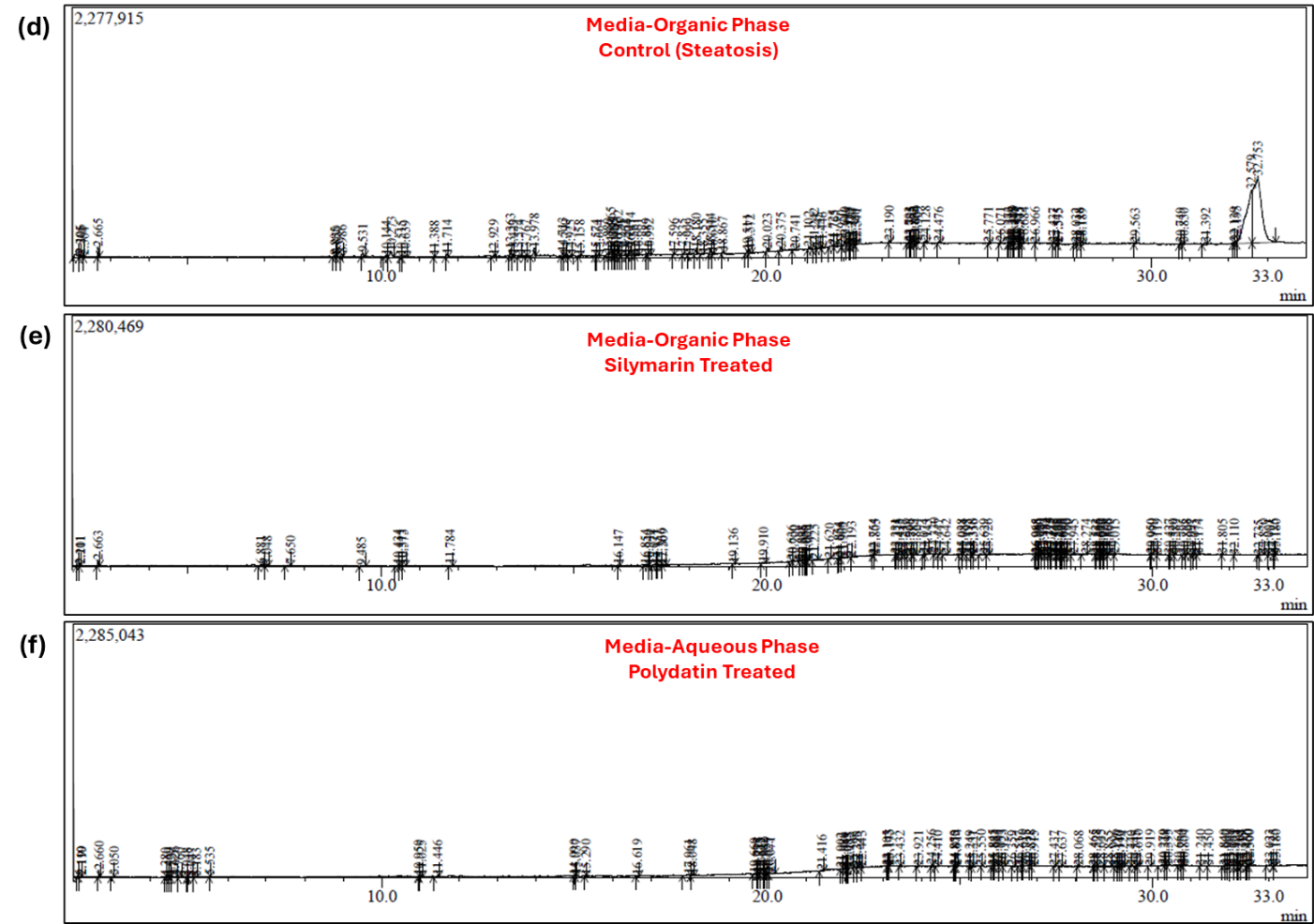
**
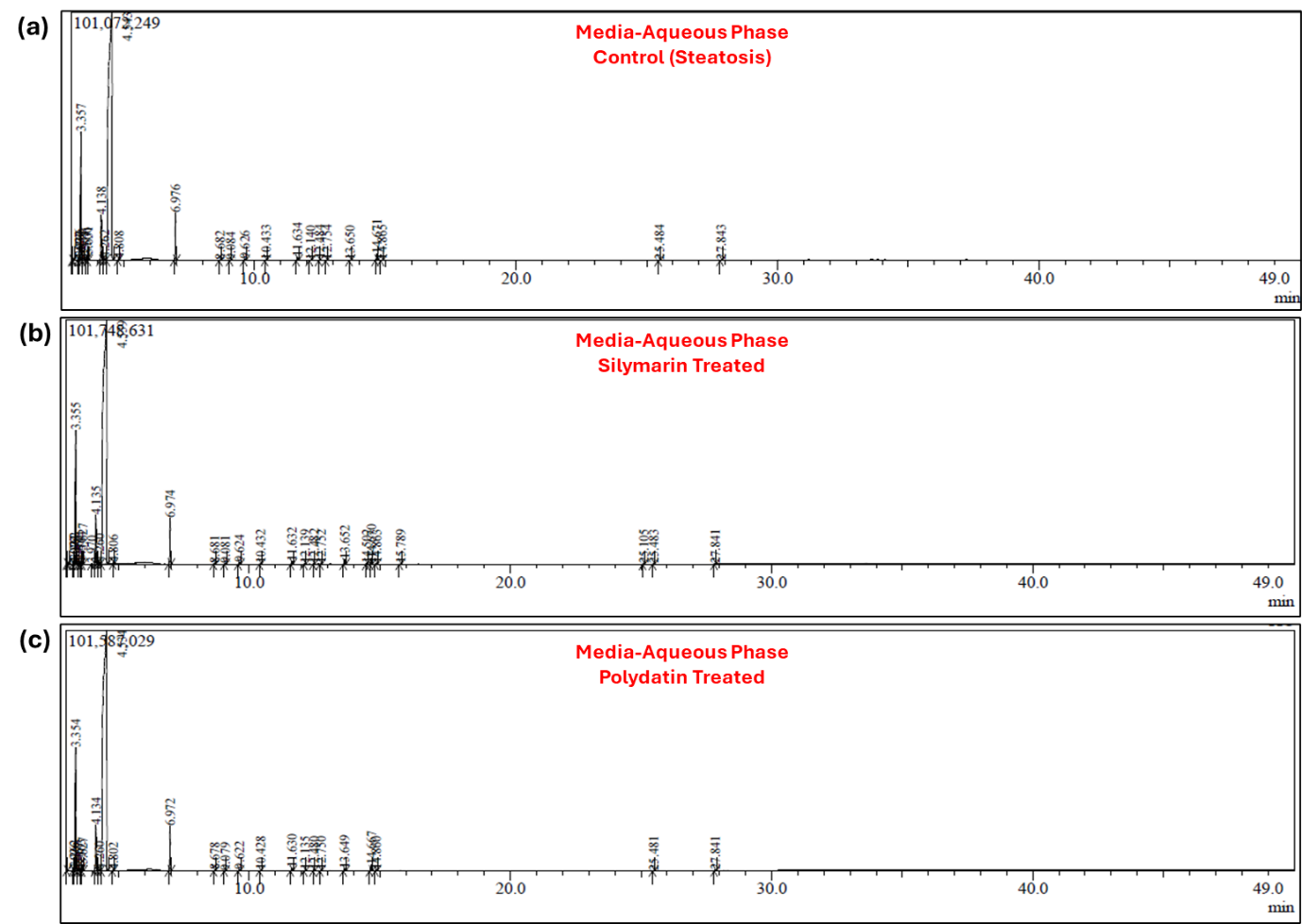


**
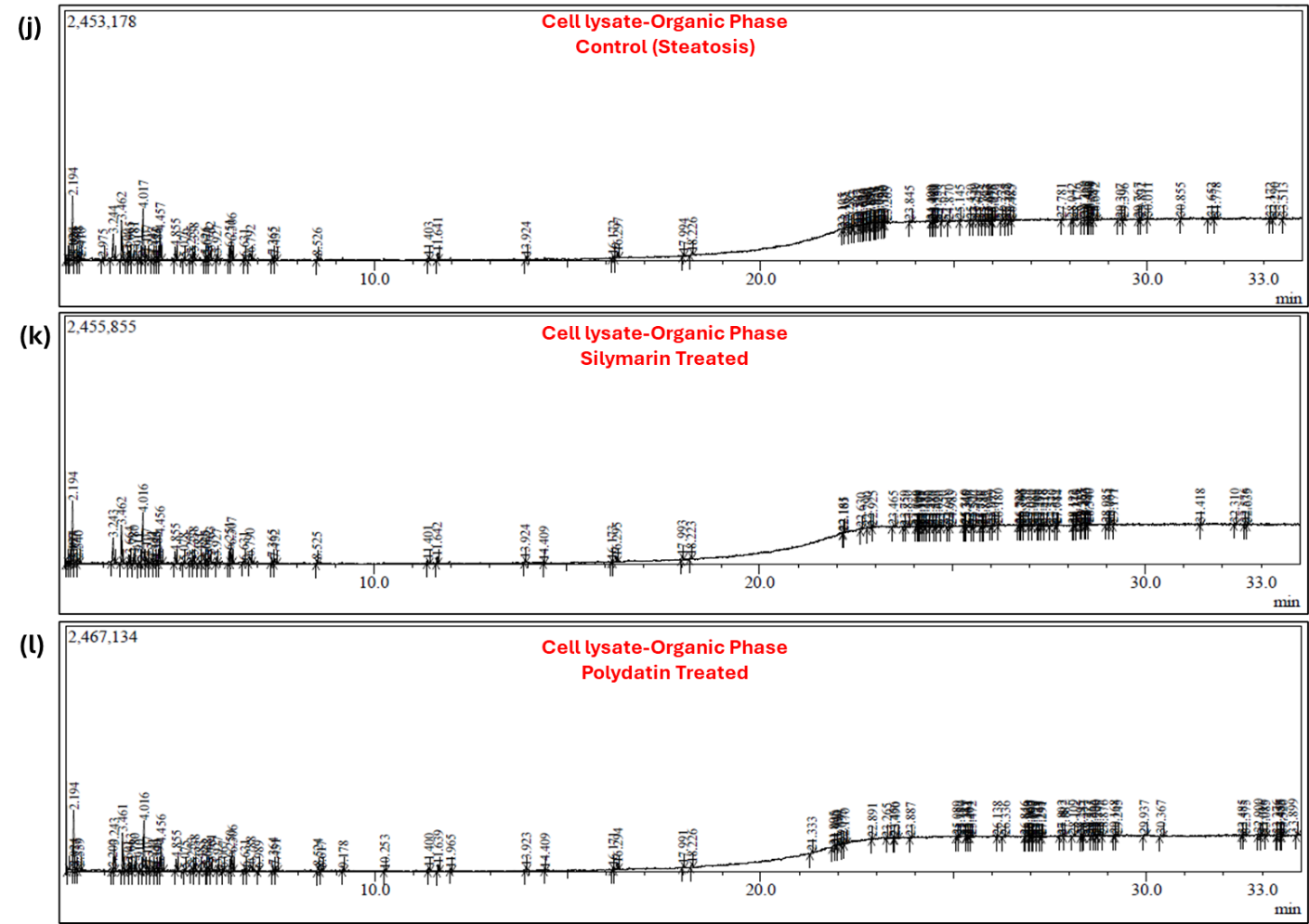

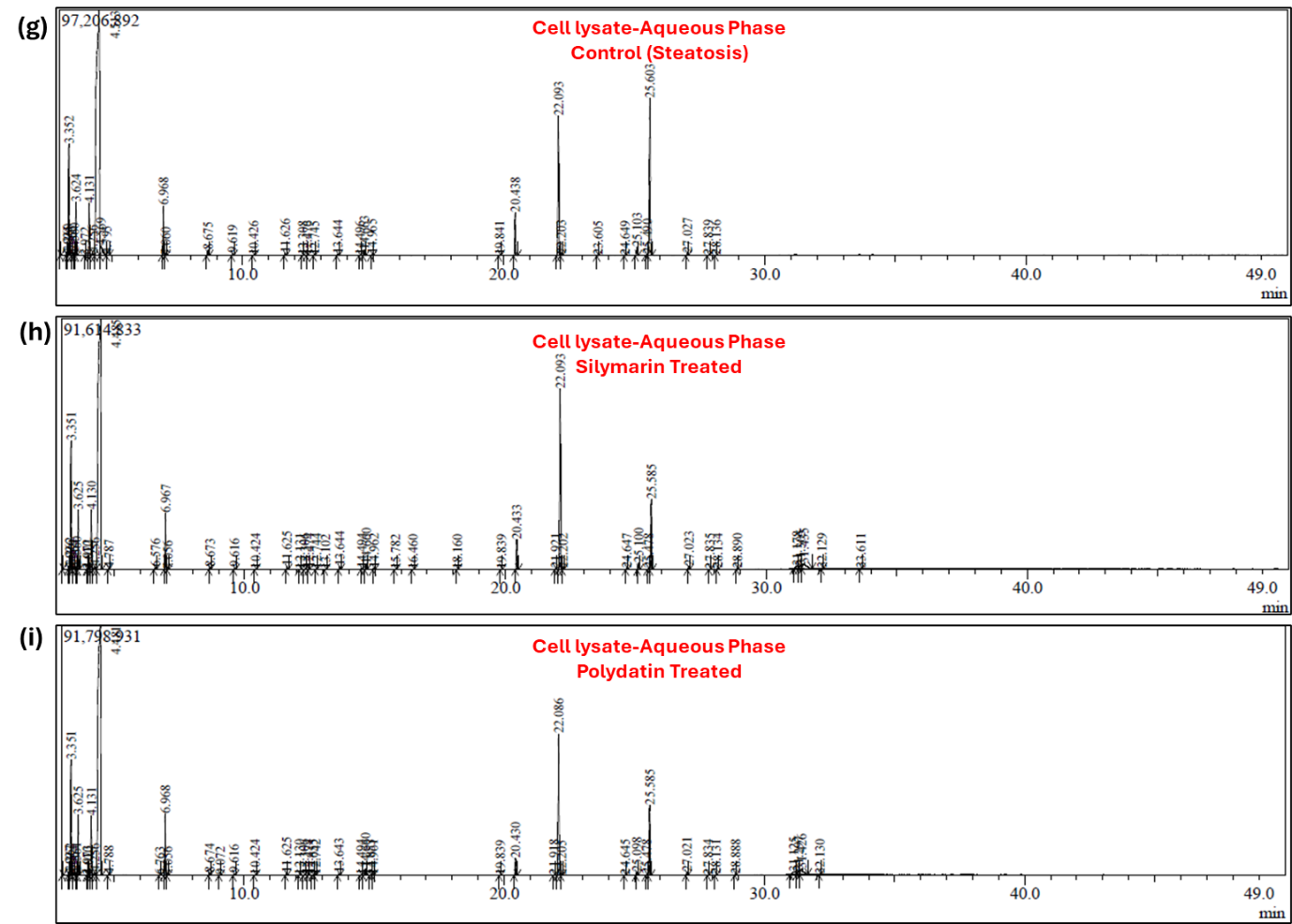
**

**Supplementary Figure 4: GC-MS chromatograms of different groups**. **a**. Media-aqueous phase (Steatotic disease model) **b**. Media-aqueous phase (Silymarin treated) c. Media-aqueous phase (Polydatin treated) **d**. Media-organic phase (Steatotic disease model) **e**. Media-organic phase (Silymarin treated) **f**. Media-organic phase (Polydatin treated) **g**. Cell lysate -aqueous phase (Steatotic disease model) **h**. Cell lysate-aqueous phase (Silymarin treated) **i**. Cell lysate-aqueous phase (Polydatin treated) **j**. Cell lysate-Organic phase (Steatotic disease model) **k**. Cell lysate-organic phase (Silymarin treated) **l**. Cell lysate -organic phase (Polydatin Treated).

**Original images of Western blots**

(A)


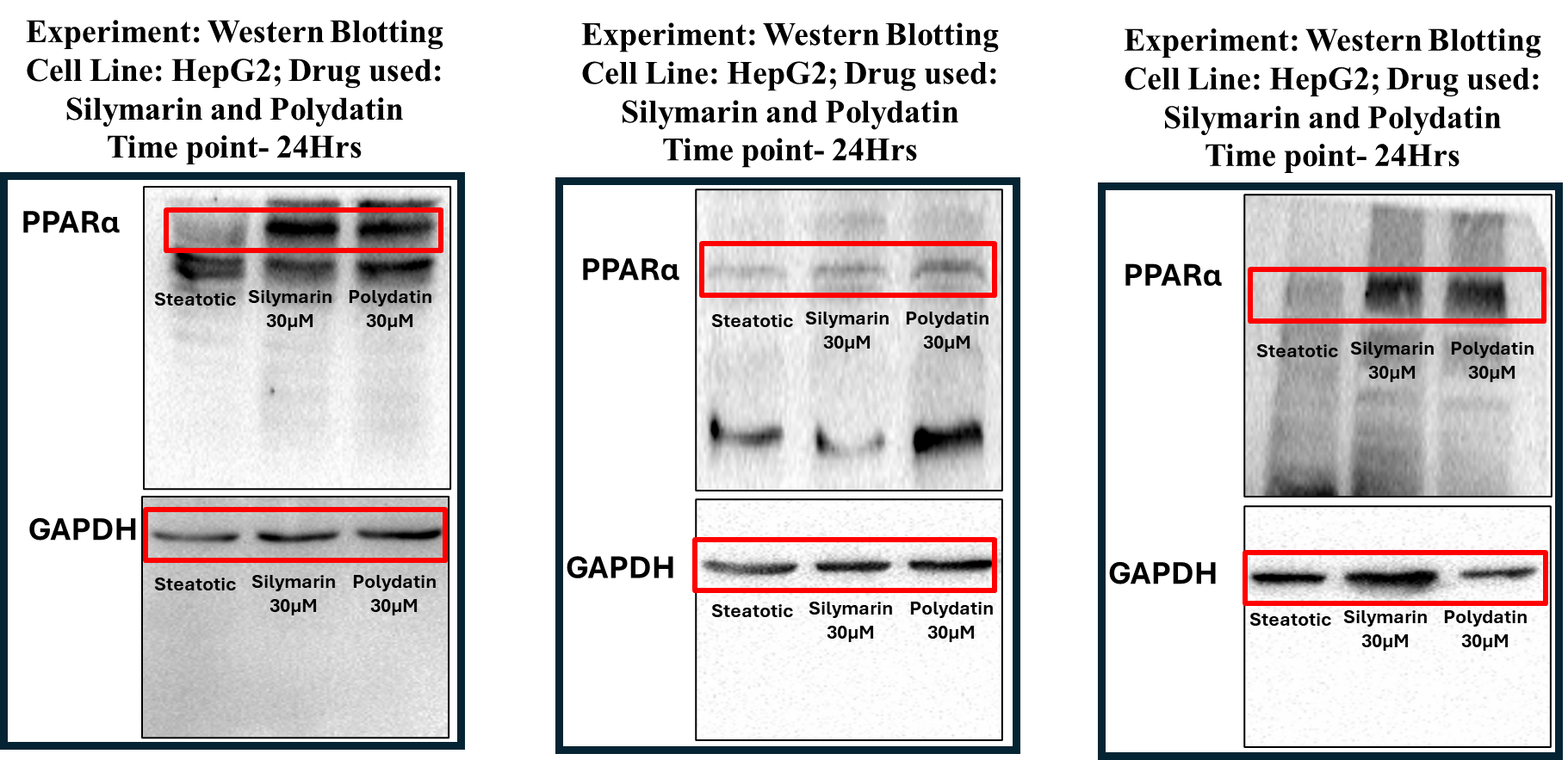


(B)


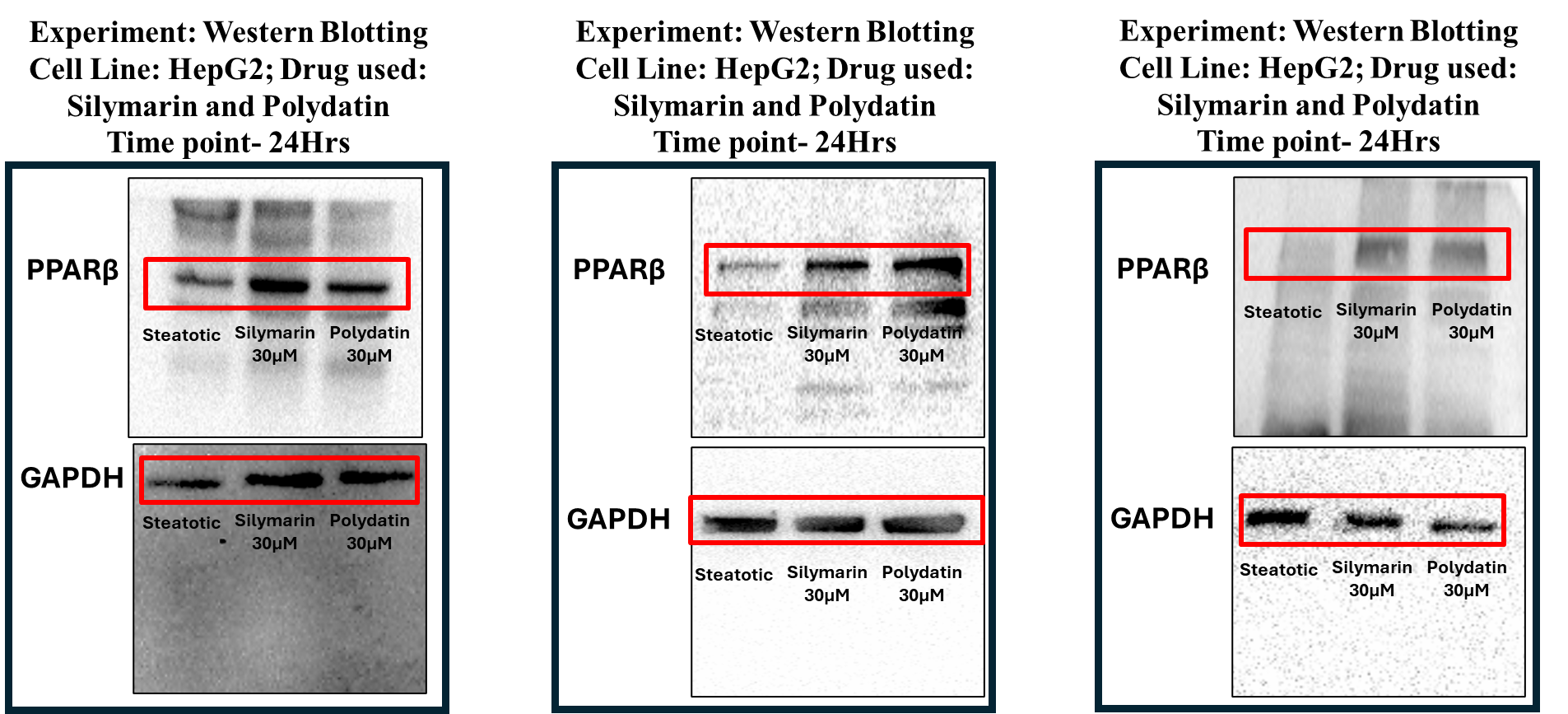


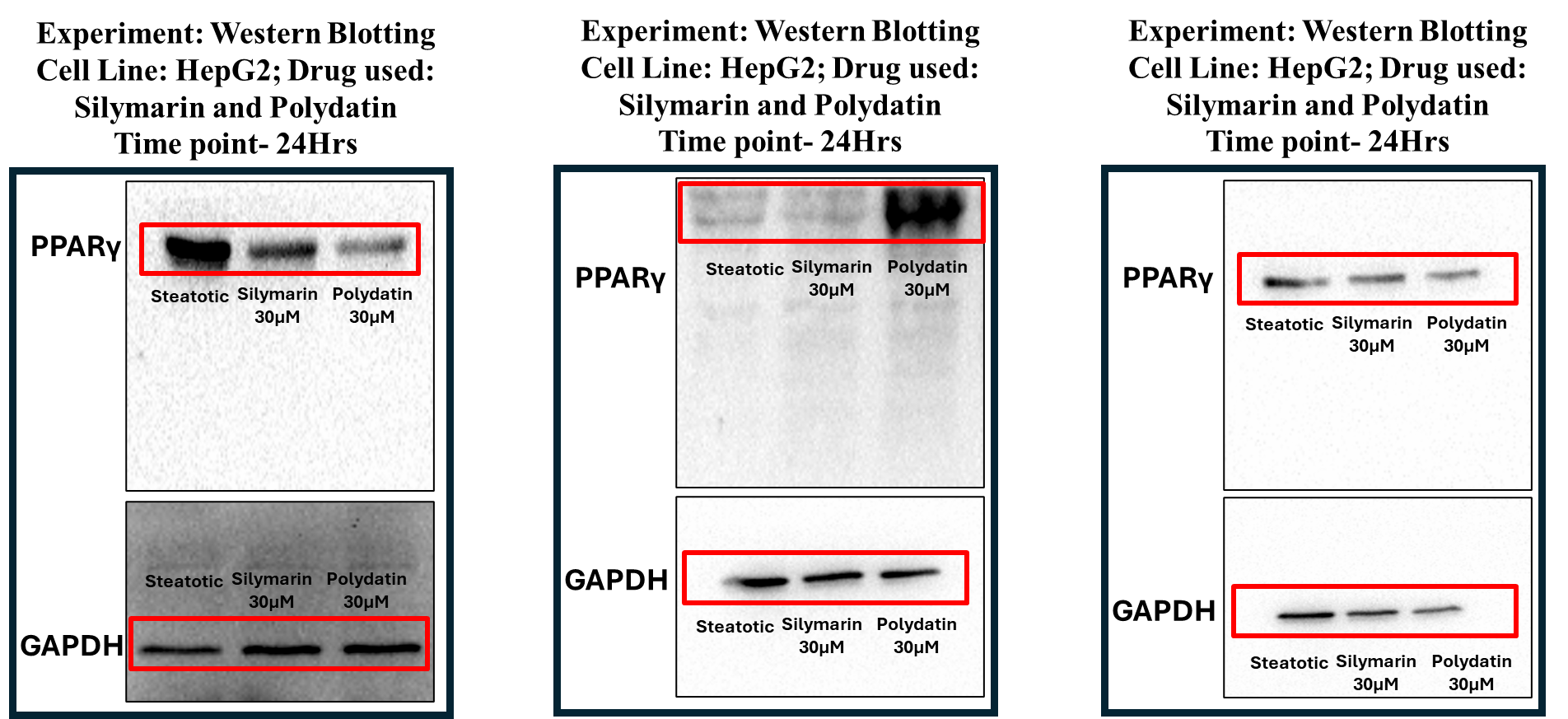

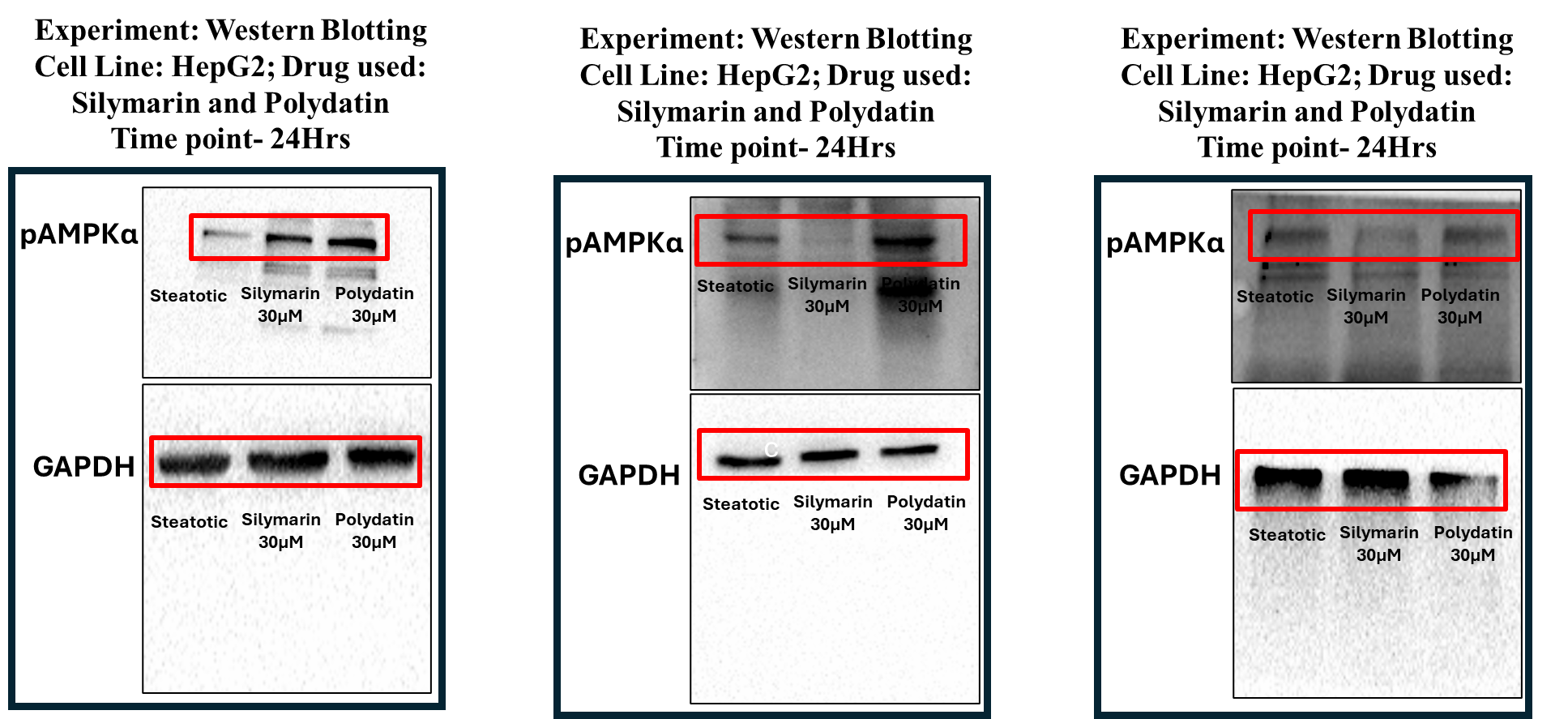

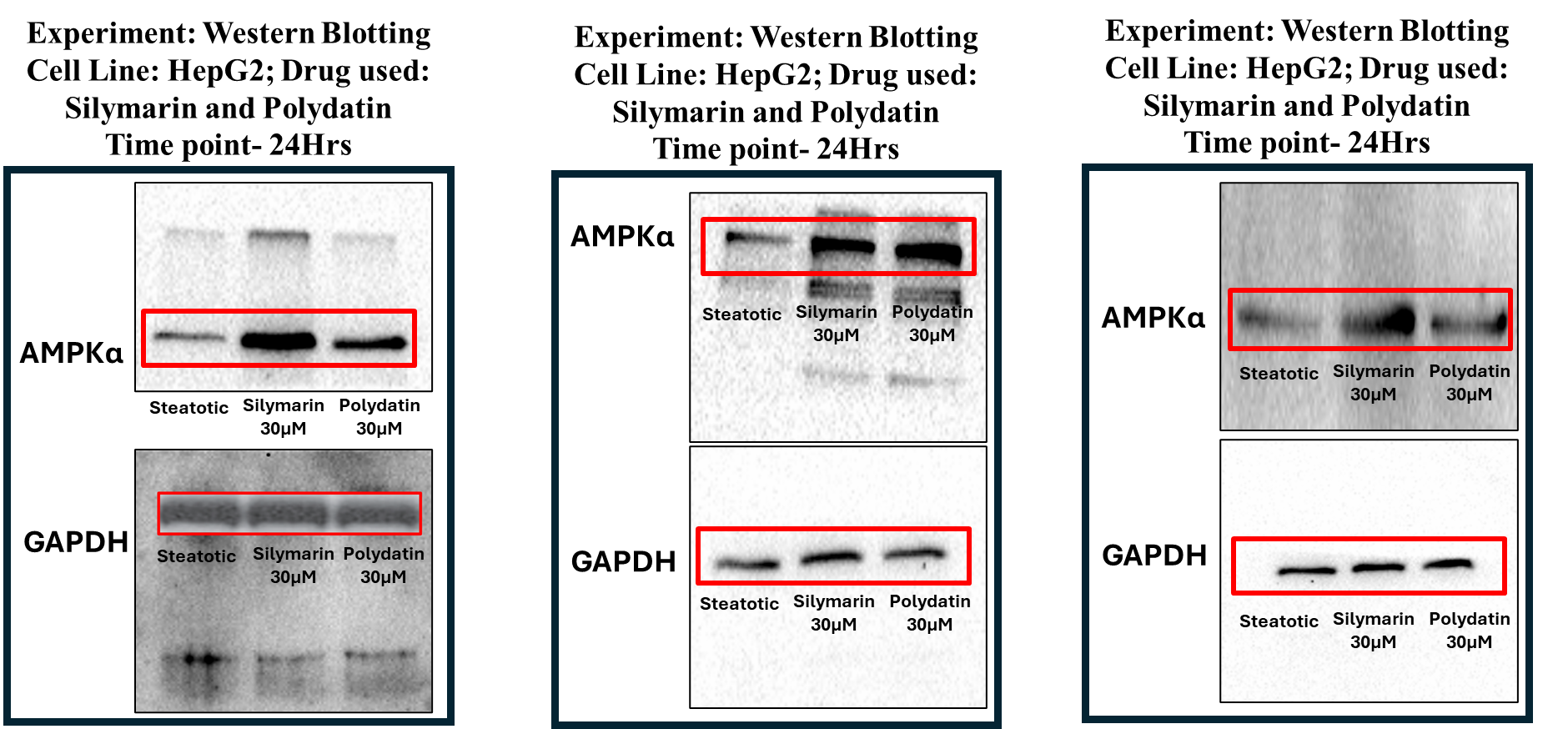


(E)

(D)

(C)


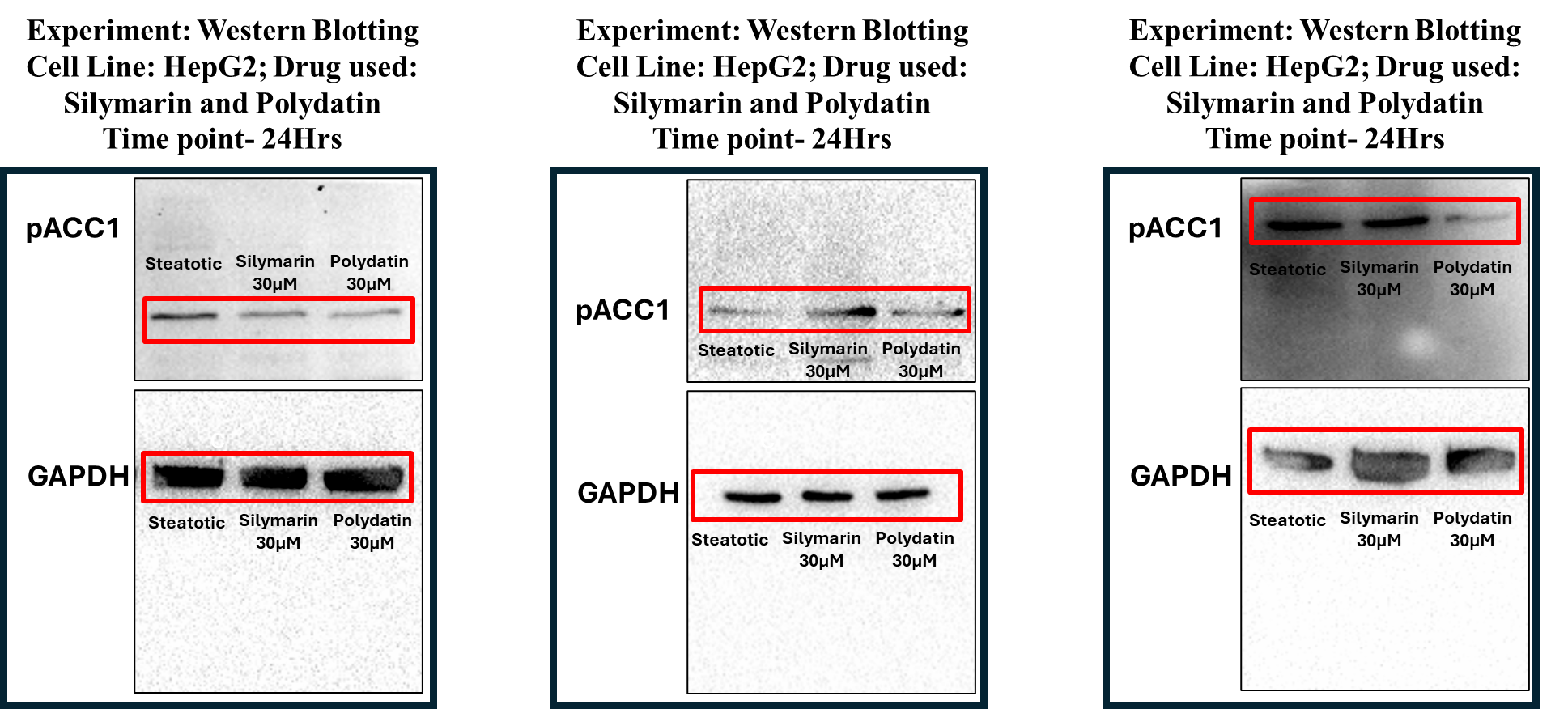

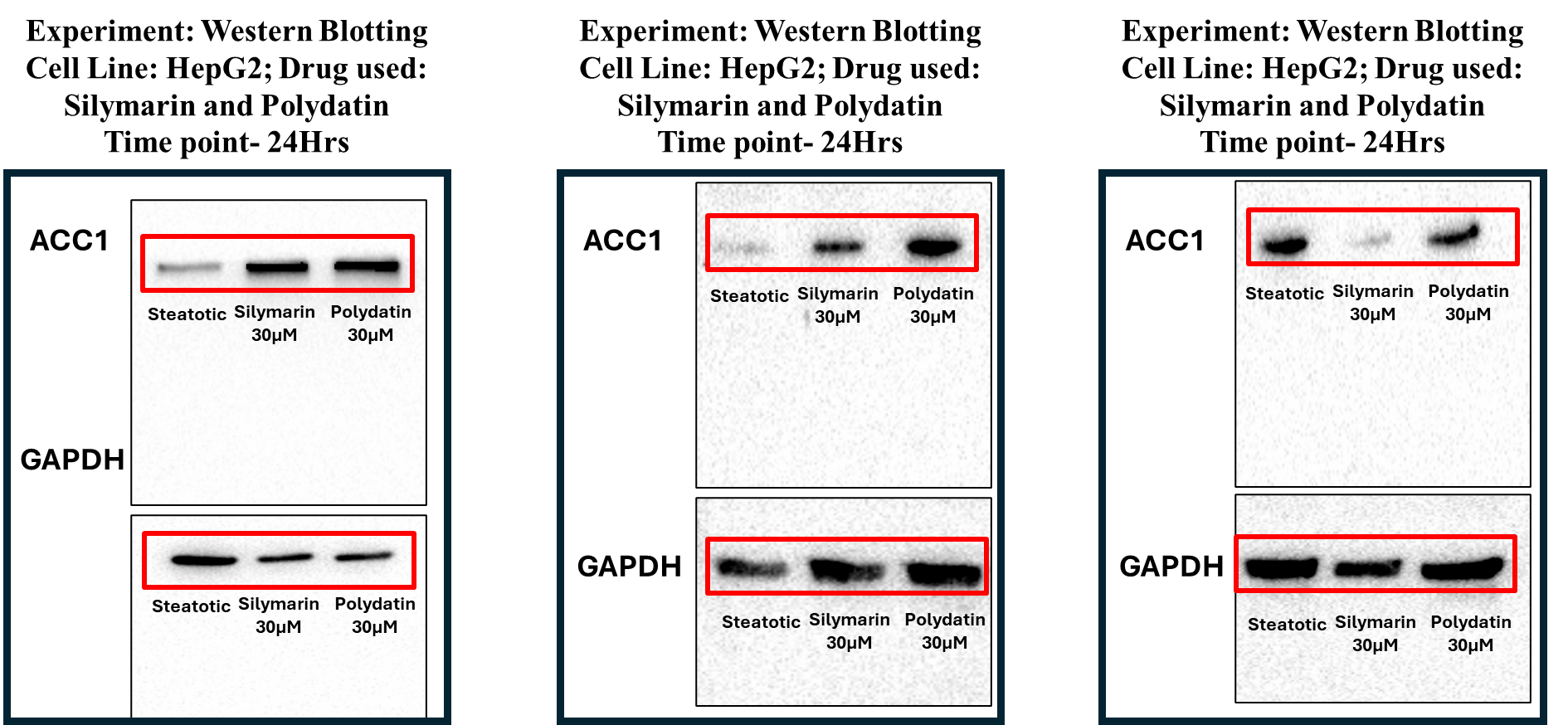

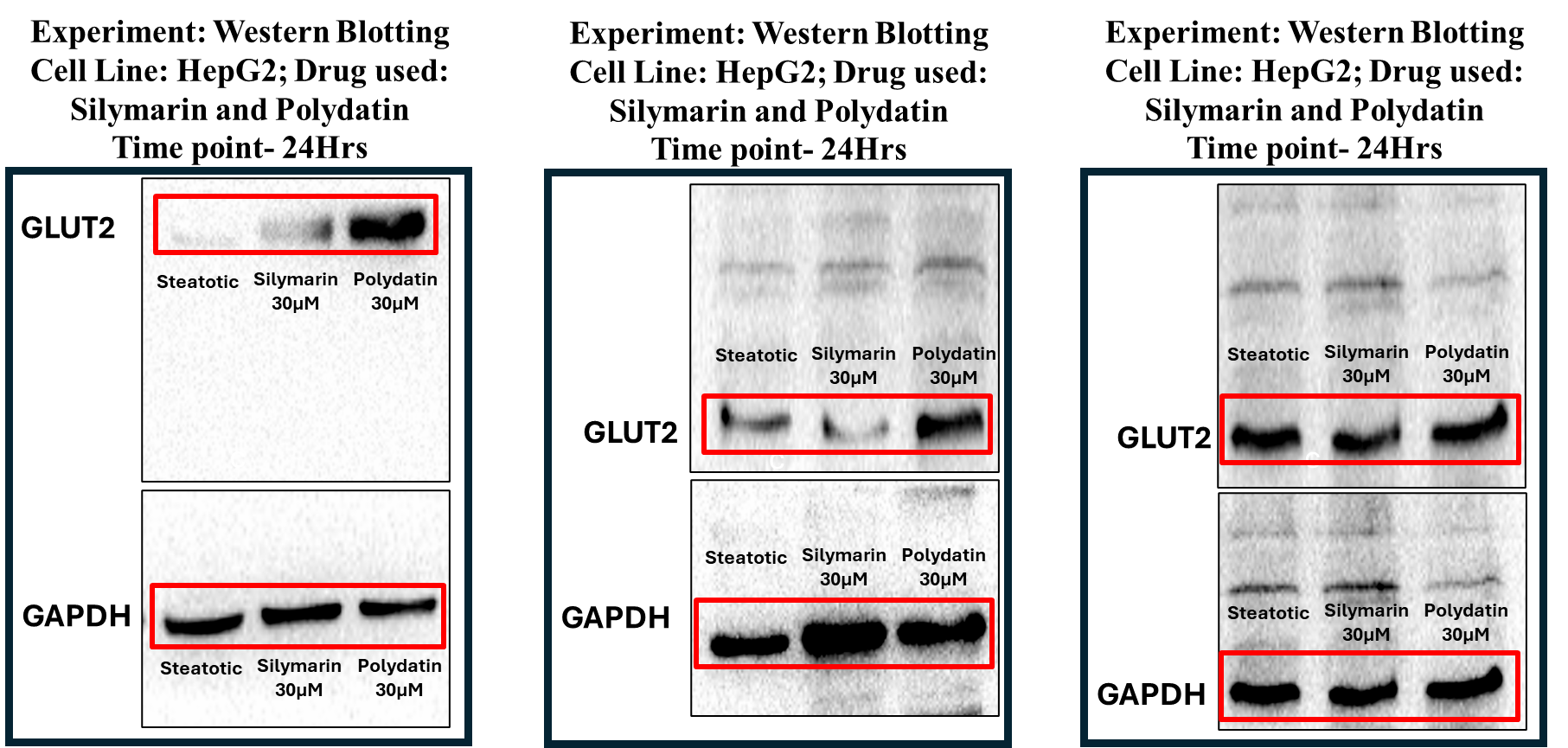


(H)

(G)

(F)

(I)


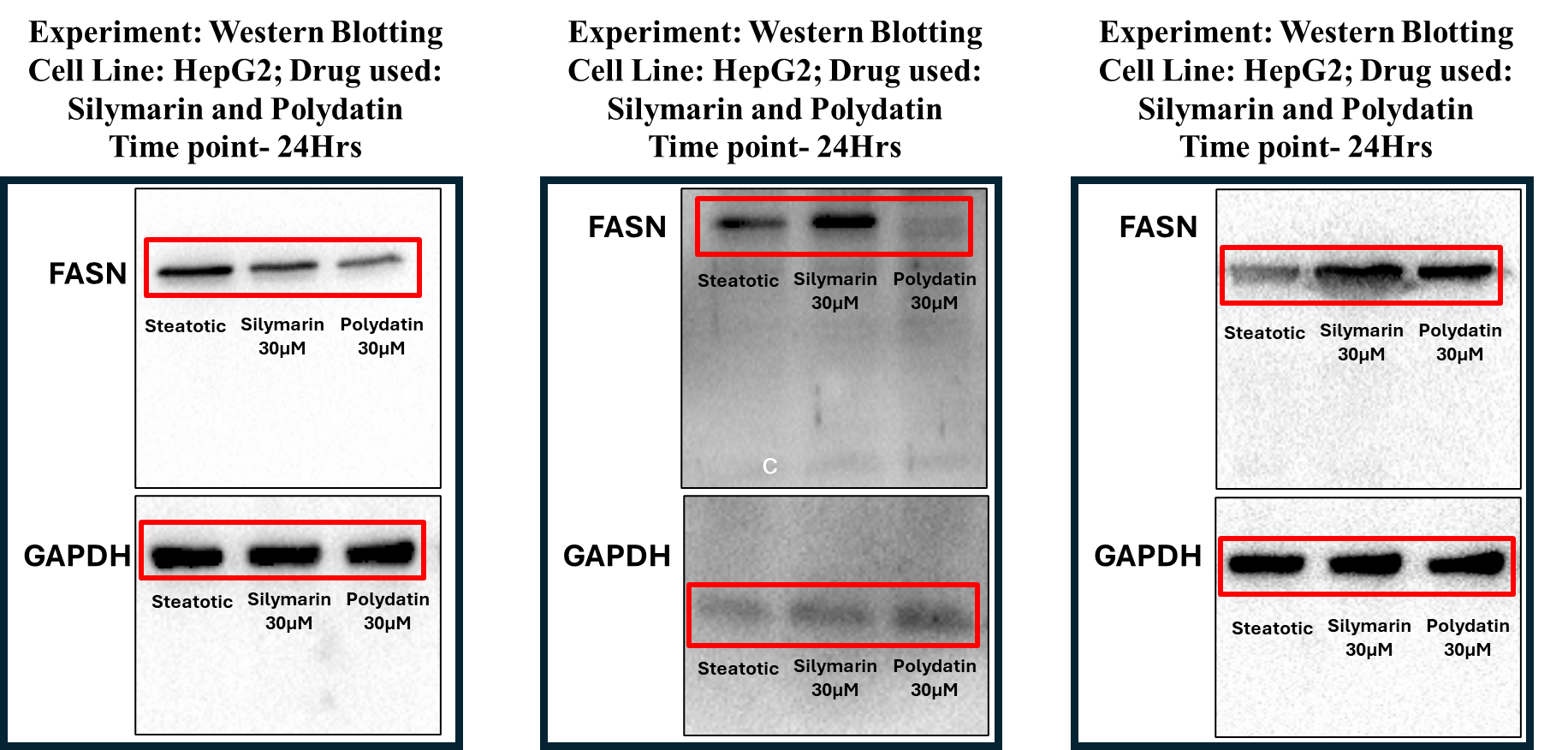


(J)


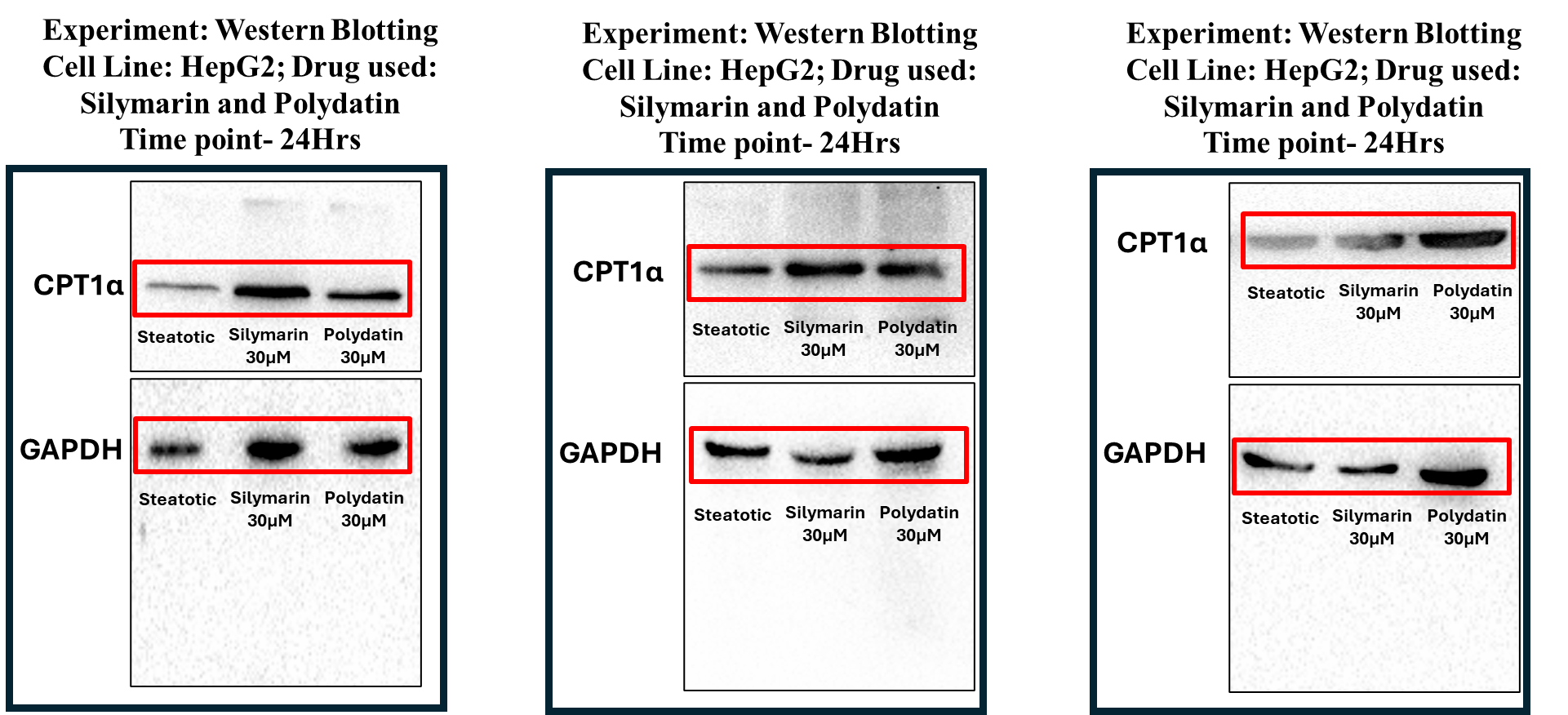


**Supplementary Figure 5: Polydatin modulates hepatic protein expression related to lipid and energy metabolism.** (A) PPARα, (B) PPARβ, (C) PPARγ, (D) AMPKα, (E) pAMPKα, (F) GLUT2, (G) ACC1, (H) p-ACC, (I) FASN, and (J) CPT1α.
